# Supplementary material for: hnRNPH1 recruits PTBP2 and SRSF3 to modulate alternative splicing in germ cells
Source: Nat Commun. 2022 Jun 23;13:3588. doi: 10.1038/s41467-022-31364-7 (PMC9226075; doi:10.1038/s41467-022-31364-7)
Supplement: Supplementary file 1 — Supplementary Information [file 41467_2022_31364_MOESM1_ESM.pdf]

Supplementary Information

hnRNPH1 recruits PTBP2 and SRSF3 to modulate alternative splicing in germ cells

Shenglei Feng[1#], Jinmei Li[1#], Hui Wen[1], Kuan Liu[1], Yiqian Gui[1], Yujiao Wen[1], Xiaoli Wang[1], Shuiqiao Yuan[1, 2,3*]

[1]Institute Reproductive Health, Tongji Medical College, Huazhong University of Science and Technology, Wuhan, Hubei 430030, China

[2]Laboratory Animal Center, Huazhong University of Science and Technology, Wuhan, Hubei 430030, China

[3]Shenzhen Huazhong University of Science and Technology Research Institute, Shenzhen, Guangdong 518057, China

Supplementary Figure 1

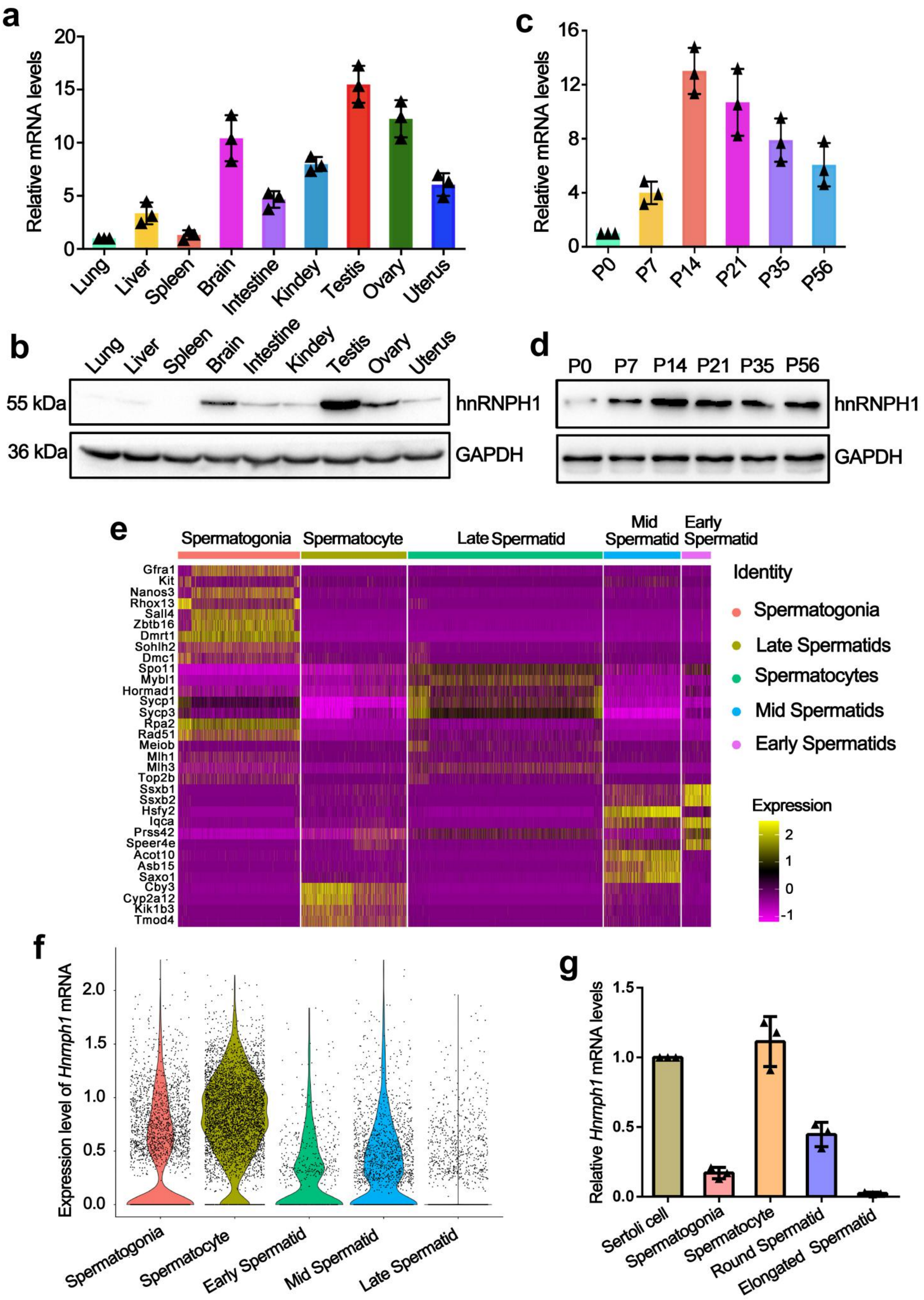

Supplementary Fig.1. Expression profiles of hnRNPH1 during spermatogenesis in mice. a RT-qPCR analyses of *Hnrnph1* mRNA levels in multiple organs from WT adult mice. Data are presented as mean ± SD, n = 3 (three biological replicates). **b** Expression of hnRNPH1 protein levels

in adult WT multiple organs by Western blot analyses. GAPDH serves as the loading control. Biologically independent mice (n=3) were examined in three separate experiments. **c** RT-qPCR analyses of *Hnrnph1* mRNA levels in developing testes at postnatal day 0 (P0), P7, P14, P21, P35, and P56. Data are presented as mean ± SD, n = 3 (three biological replicates). **d** Expression of hnRNPH1 protein in developing testes by Western blot analyses. GAPDH serves as the loading control. Biologically independent mice (n=3) were examined in three separate experiments. **e** Differentially expressed genes of each sample were conducted using a Wilcoxon Rank Sum test with *p*-value <0.05, log FC. threshold=0.5. Cells types were allocated to each cluster using the known marker genes described previously (PMID: 30404016). Biologically independent mice (n = 3) for each genotype were examined. **f** Expression of Hnrnph1 in different types of germ cells based on single-cell RNA-seq (PMID: 30404016). **g** RT-qPCR analyses of *Hnrnph1* mRNA levels in different types of germ cells isolated from WT adult mice. Data are presented as mean ± SD, n = 3 (three biological replicates). Source data are provided as a source data file.

Supplementary Figure 2

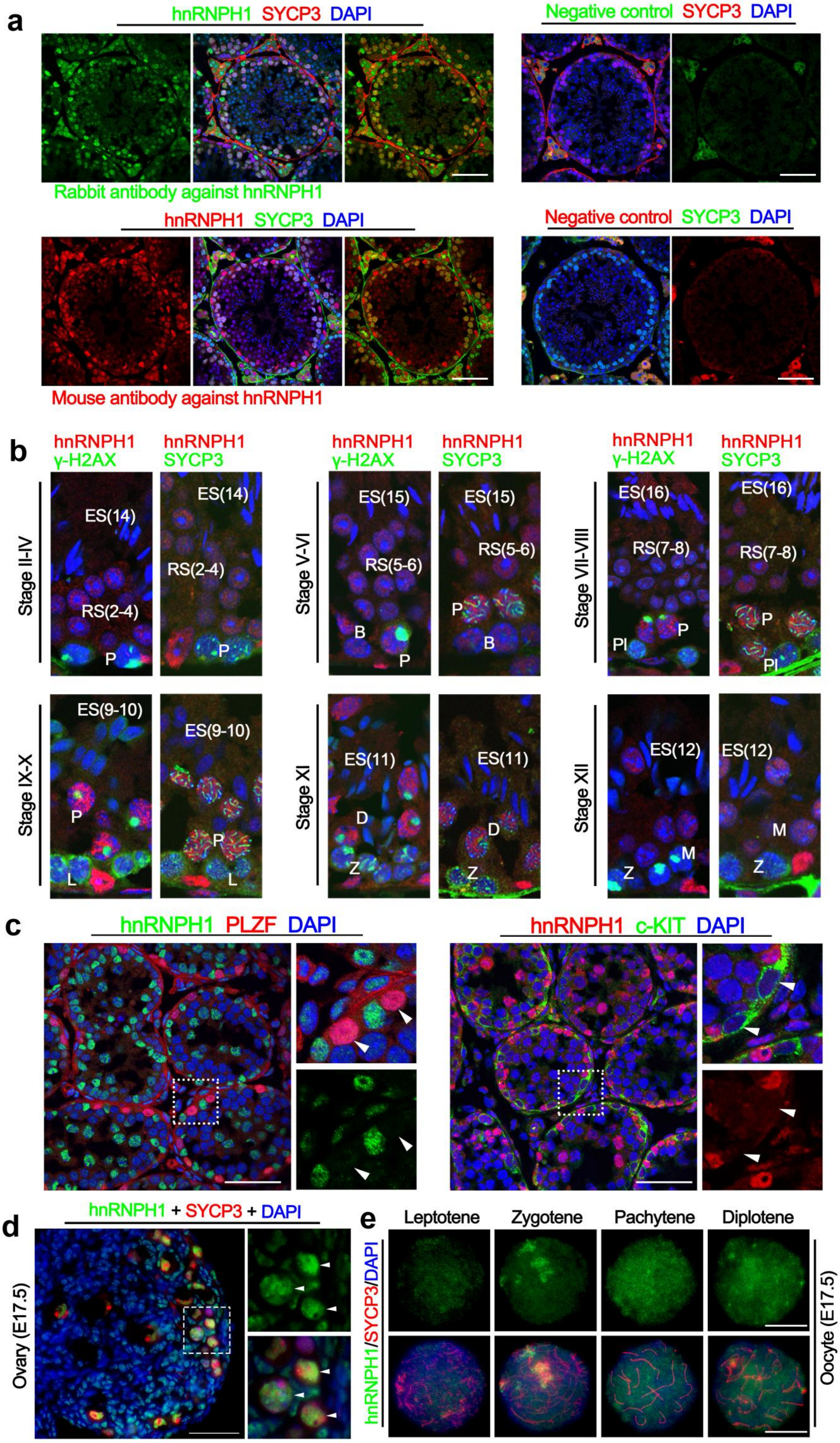

Supplementary Fig.2. The localization of hnRNPH1 in mouse germ cells. a Co-immunofluorescence staining of SYCP3 and different sources of hnRNPH1 antibodies on WT testis sections are shown. The right panels are negative control with no anti-hnRNPH1. Nuclei were stained with DAPI. Scale bars = 50μm. **b** Co-immunofluorescence staining of anti-hnRNPH1 with anti-γH2AX or anti-SYCP3 on WT adult testis sections are shown. Nuclei were stained with DAPI. Scale bars = 50μm. **c** Co-immunofluorescence staining of anti-hnRNPH1 with anti-PLZF or anti-c-KIT on staged WT testis sections at P14 are shown. Nuclei were stained with DAPI. Scale bars = 50μm. **d** Co-immunofluorescence staining of hnRNPH1 with SYCP3 in ovaries from WT mice at embryonic day 17.5 (E17.5) is shown. Scale bar = 50μm. **e** Double immunostaining with hnRNPH1 and SYCP3 on surface-spread oocytes from WT mice at E17.5 are shown. Biologically independent mice (n=3) were examined in three separate experiments (a-e). Scale bars = 5μm.

Supplementary Figure 3

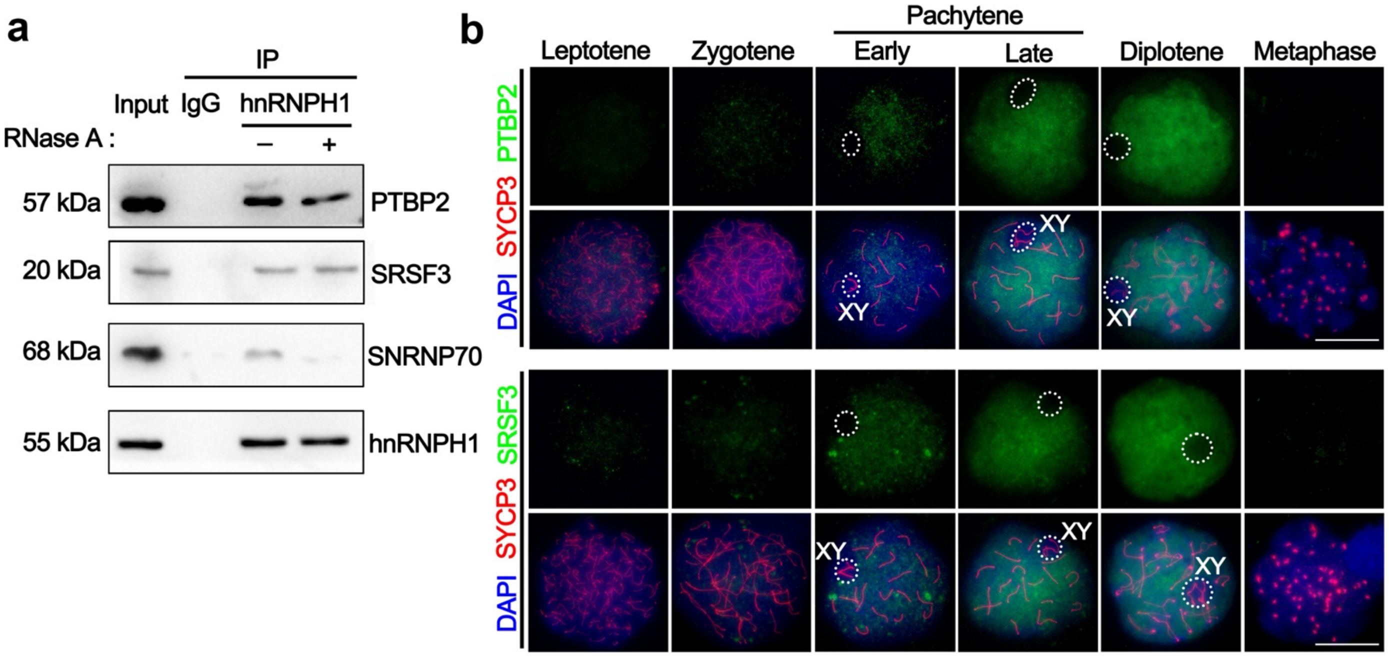

Supplementary Fig.3. The interaction between hnRNPH1 and its bound proteins and their localization in spermatocytes. a Immunoprecipitation of hnRNPH1 on WT adult mouse testis lysate with or without RNase A treatment followed by western blot using indicated antibodies are shown. IgG was used as a negative control. Biologically independent mice (n=3) were examined in three separate experiments. **b** Co-immunofluorescence staining of SYCP3 with PTBP2 (upper) and SRSF3 (lower) on surface-spread spermatocytes from WT mice are shown. The dotted lines outline XY-body. Biologically independent mice (n = 3) for each genotype were examined. Scale bar = 5μm. Source data are provided as a source data file.

Supplementary Figure 4

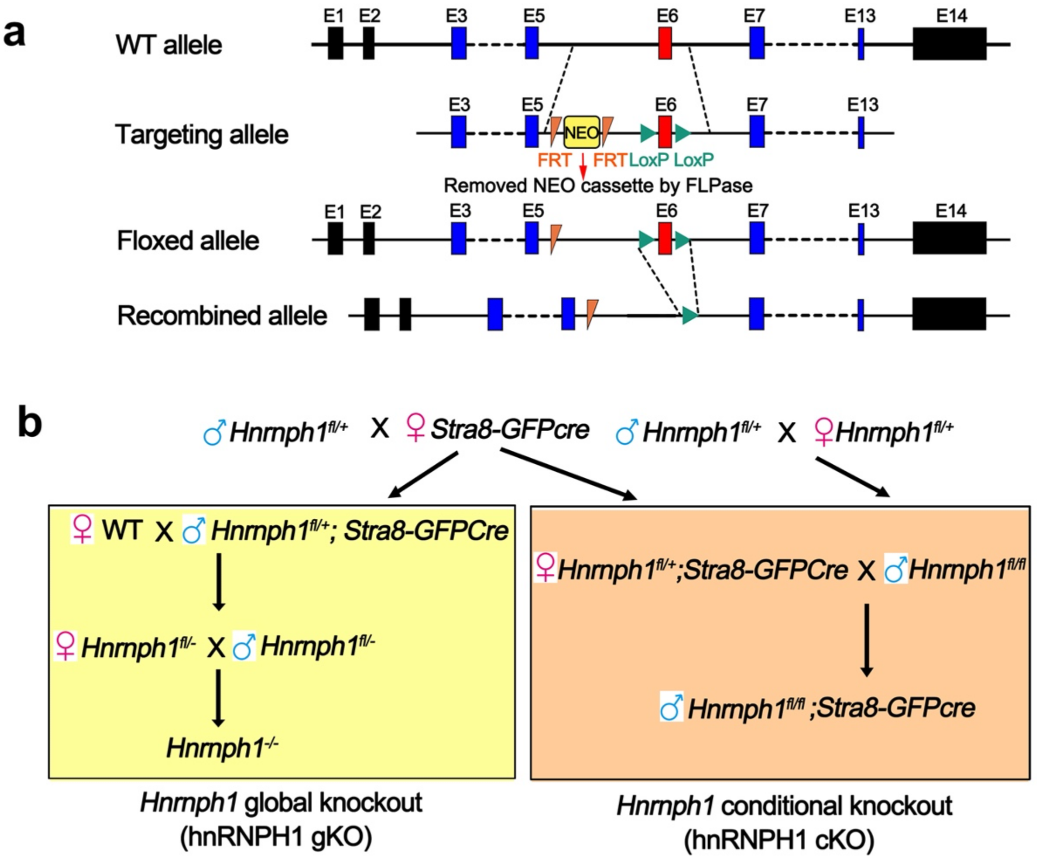

c

	WT	HT	KO	Total
4wks	58	132	0	190
E14.5	33	59	0	92
E13.5	47	88	10	145
E12.5	38	81	22	141
E10.5	54	97	43	194
E9.5	68	133	71	272

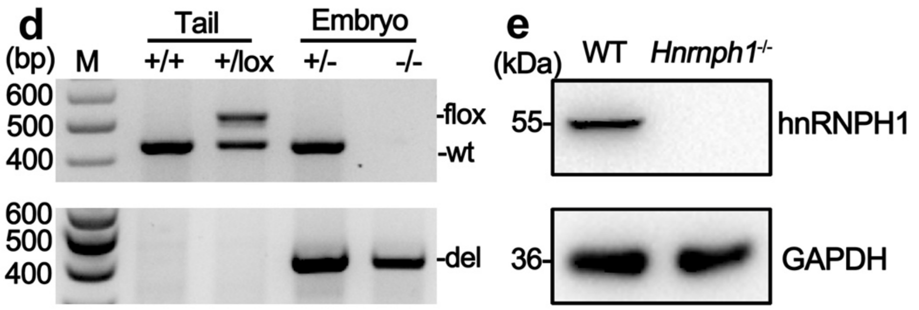

Supplementary Fig.4. Generation of both conventional and conditional *Hnrnph1* knockout mouse models. a Schematic diagram of the targeting strategy used to generate floxed *Hnrnph1* allele by homologous recombination in mouse embryonic stem cells. Exons 6 was deleted after Cre- mediated recombination. **b** Schematic diagram of the generation of both global and germline conditional *Hnrnph1* knockout mice. **c** The number of embryonic offspring with different genotypes produced by mating heterozygous *Hnrnph1* male and female mice are shown. **d** PCR-based genotyping of the mouse tails and E12.5 embryos is shown. Biologically independent mice (n=3) were examined in three separate experiments. **e** Western blotting of the hnRNPH1 in the lysates of WT and *hnRNPH1*$^{-/-}$ embryos at E12.5. GAPDH was blotted as a loading control. Biologically independent mice (n = 3) for each genotype were examined. Source data are provided as a source data file.

Supplementary Figure 5

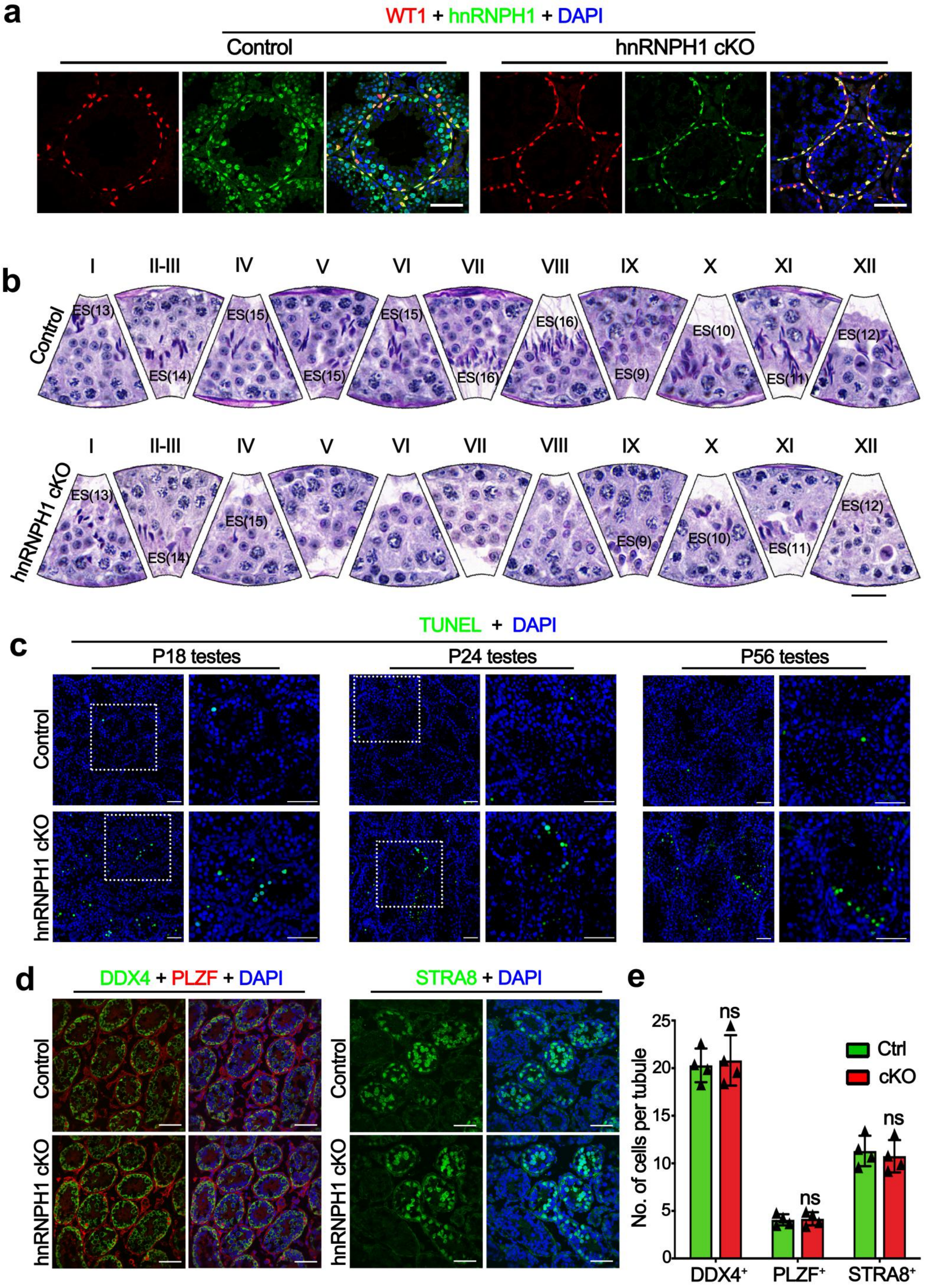

Supplementary Fig.5. Conditional deletion of hnRNPH1 in male germ cells causes defective spermatogenesis. a Co-immunofluorescence staining of anti-hnRNPH1 with anti-WT1 on testis sections from control and hnRNPH1 cKO adult mice are shown. Nuclei were stained with DAPI. Biologically independent mice (n=3) were examined in three separate experiments. Scale bars = 50μm. **b** Periodic acid-Schiff (PAS) staining of staged testis sections from control and hnRNPH1 cKO mice at P56. Scale bar = 25μm. **c** TUNEL staining of testes from control and hnRNPH1 cKO mice at P18, P24, and P56. The DNA was stained with DAPI. Biologically independent mice (n = 3) for each genotype were examined. Scale bars = 50μm. **d** Immunofluorescence staining of DDX4/PLZF (*left*) and STRA8 (*right*) on testis sections from control and hnRNPH1 cKO mice at P10. The DNA was stained with DAPI. Scale bars = 50μm. **e** The quantification of $DDX4^{+}$, $PLZF^{+}$, and $STRA8^{+}$ cells per tubule for (d). Data are presented as mean ± SD, n = 4 (four biological replicates), ns, not significant. Source data are provided as a source data file.

Supplementary Figure 6

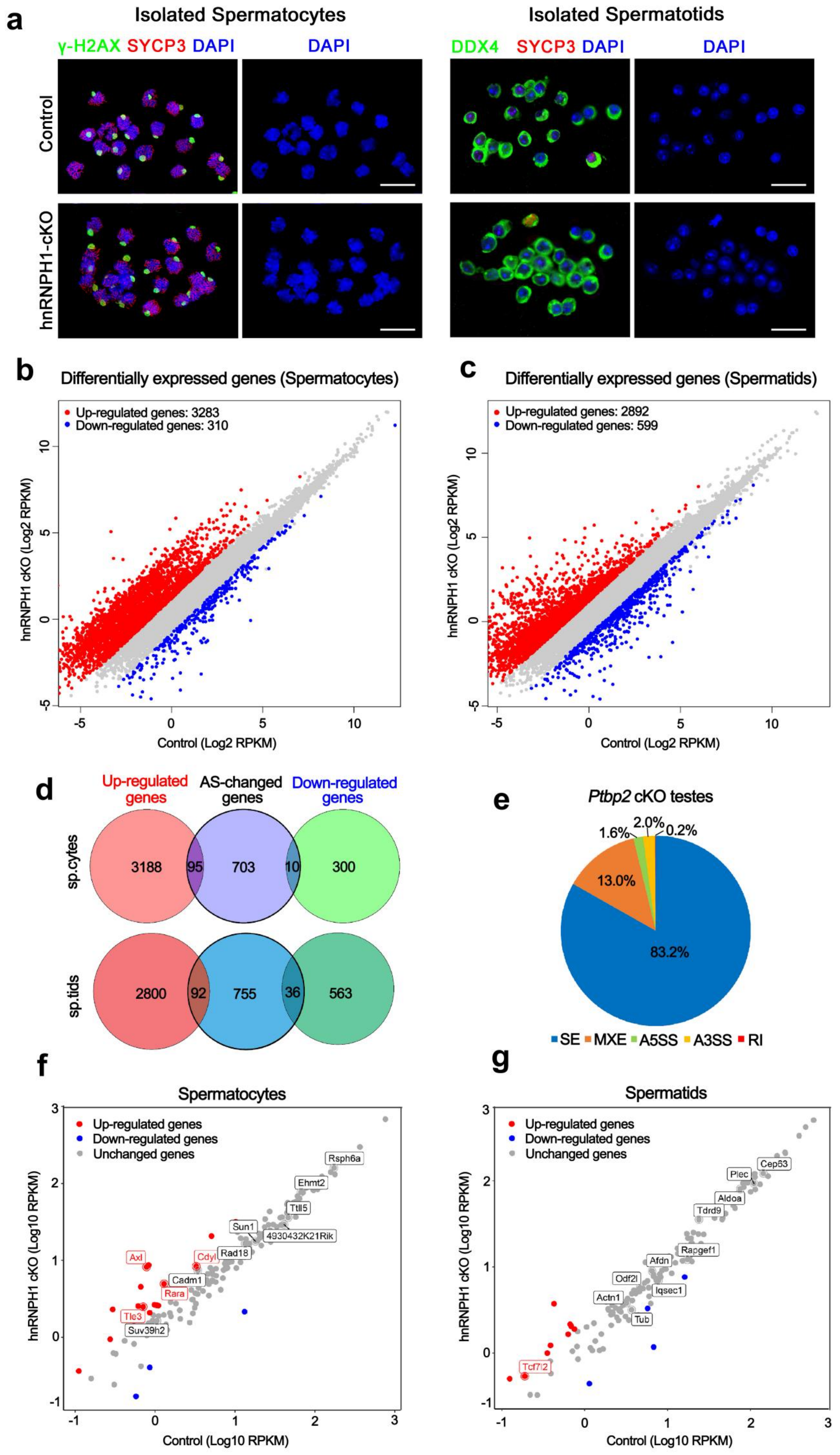

Supplementary Fig.6. Analysis of RNA-seq data and the comparison of hnRNPH1- and PTBP2-regulated genes. a The left panels showing co-immunofluorescence staining of anti-γH2AX with anti-SYCP3 on isolated spermatocytes from control and hnRNPH1 cKO mice. Nuclei were stained with DAPI. Biologically independent mice (n = 3) for each genotype were examined. Scale bars = 20μm. The right panels showing co-immunofluorescence staining of anti-DDX4 with anti-SYCP3 on isolated spermatids from control and hnRNPH1 cKO mice. Nuclei were stained with DAPI. Scale bars = 20μm. **b-c** Volcano plot showing the differentially expressed genes determined by RNA-seq analysis of hnRNPH1 cKO spermatocytes (b) and spermatids (c). **d** Venn diagrams showing overlap of splicing-changed genes, up-regulated genes, and down-regulated genes in hnRNPH1 cKO spermatocytes (upper) and spermatids (lower). **e** The pie chart represents the distribution of regulated splicing events among different types of splicing events in *Ptbp2* cKO testes. **f-g** Volcano plots depicting the differentially expressed genes with the same abnormal AS events caused by hnRNPH1 and PTBP2 ablation in spermatocytes (f) and spermatids (g).

Supplementary Figure 7

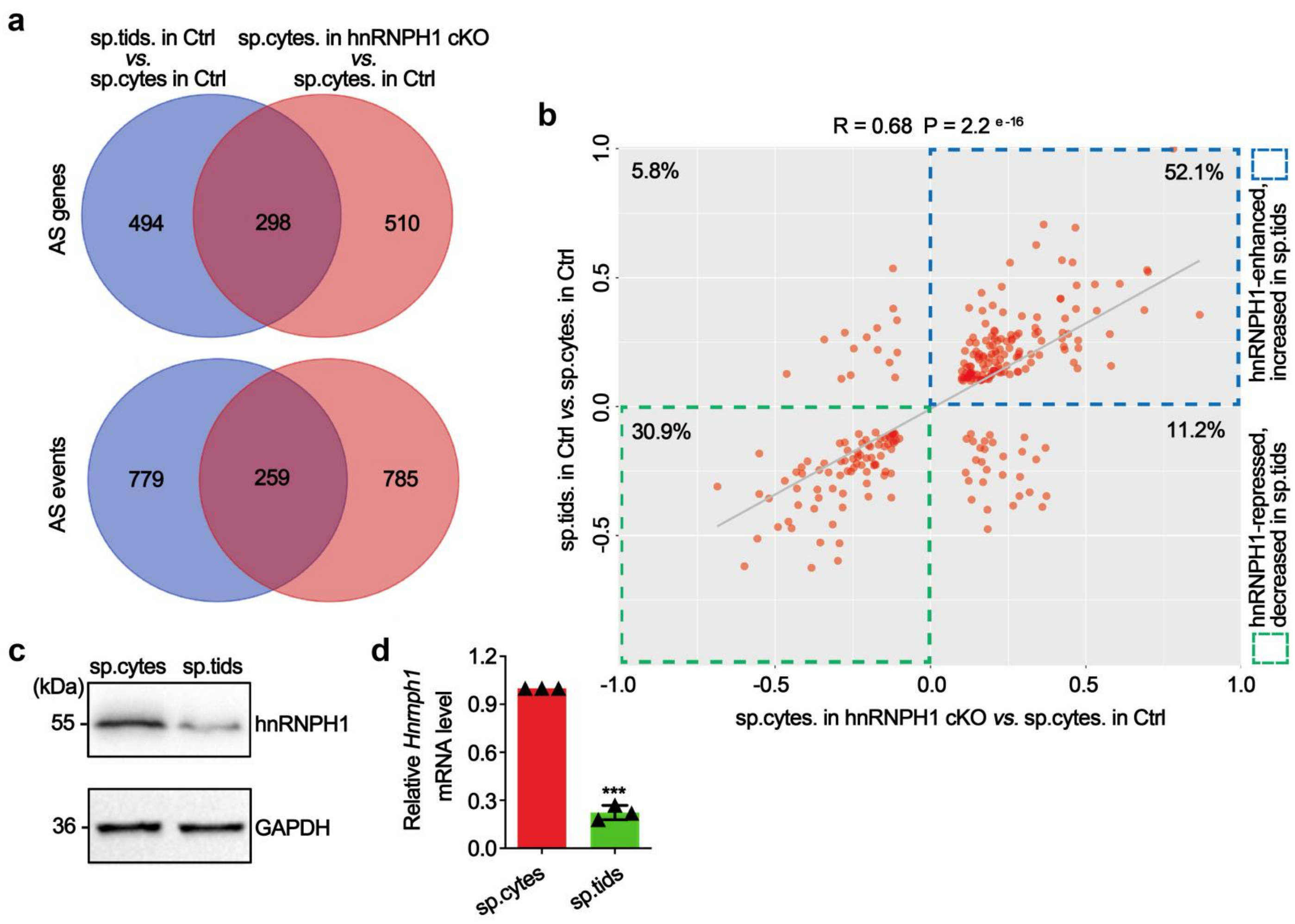

Supplementary Fig.7. AS events that occurred during the transition from spermatocytes to spermatids are related to the hnRNPH1 dynamic expression. a Venn diagrams showing the overlaps of AS genes (upper) and events (down) in the indicated comparisons. **b** Distribution of ΔPSI values for genes with AS events that abnormally appear in hnRNPH1 cKO spermatocytes and also normally occur during the transition from spermatocytes to round spermatids. **c** Expression of hnRNPH1 protein in purified spermatocytes and spermatids isolated from P25 WT mice by Western blot analyses. GAPDH serves as the loading control. **d** RT-qPCR analyses of *Hnrnph1* mRNA levels in purified spermatocytes and spermatids. Data are presented as mean ± SD, n = 3 (three biological replicates). A two-sided Student's *t*-test was performed, ***$p = 8 \times 10^{-6}$. Source data are provided as a source data file.

Supplementary Figure 8

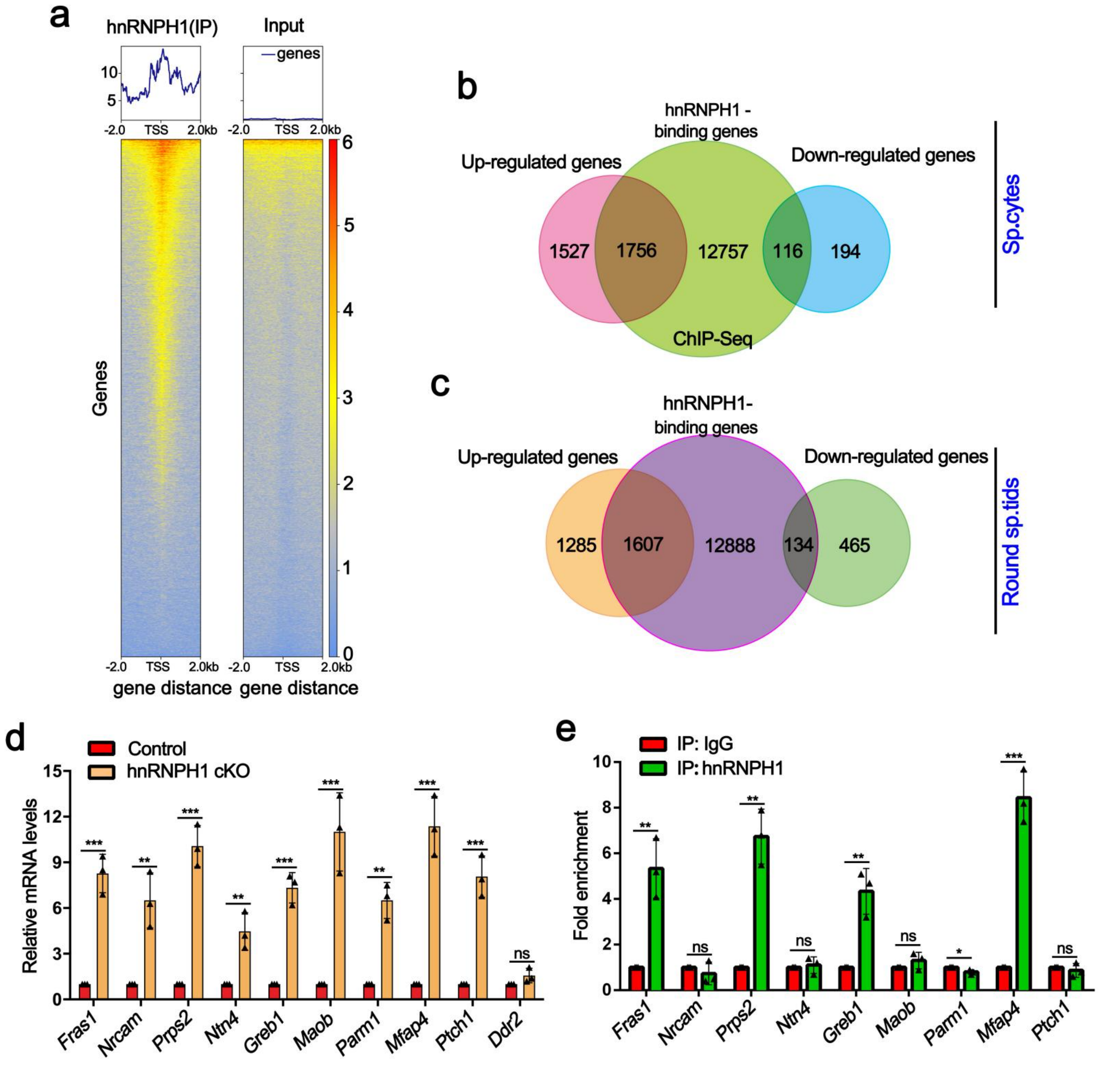

Supplementary Fig.8. Analyses of hnRNPH1 binding to the promoter of its targeted genes in germ cells. a Heatmap of hnRNPH1 binding peaks near the promoter and the transcription start site (TSS). **b-c** Venn diagrams showing overlap of hnRNPH1-binding genes and differentially expressed genes in hnRNPH1 cKO spermatocytes (b) and round spermatids (c). **d** RT-qPCR analyses of 10 up-regulated genes with the highest fold-change for qPCR verification. Data are presented as mean ± SD, n = 3 (three biological replicates). A two-sided Student's *t*-test was performed, left to right: ****p* =0.0006, ***p* =0.0062, ****p* =0.0003, ***p* =0.0080, ****p* =0.0004, ****p* =0.0025, ***p* =0.0013, ****p* =0.0008, ****p* =0.0008, ns, not significant. **e** Histograms showing ChIP-qPCR analyses of selected 9 genes precipitated by anti-hnRNPH1 antibody and control IgG. ChIP-qPCR was performed using purified germ cells. Data are presented as mean ± SD, n = 3 (three biological replicates). A two-sided Student's *t*-test was performed, left to right: ***p* =0.0045, ***p* =0.0012, ***p* =0.0045, **p* =0.0257, ****p* =0.0004, ns, not significant. Source data are provided as a source data file.

Supplementary Figure 9

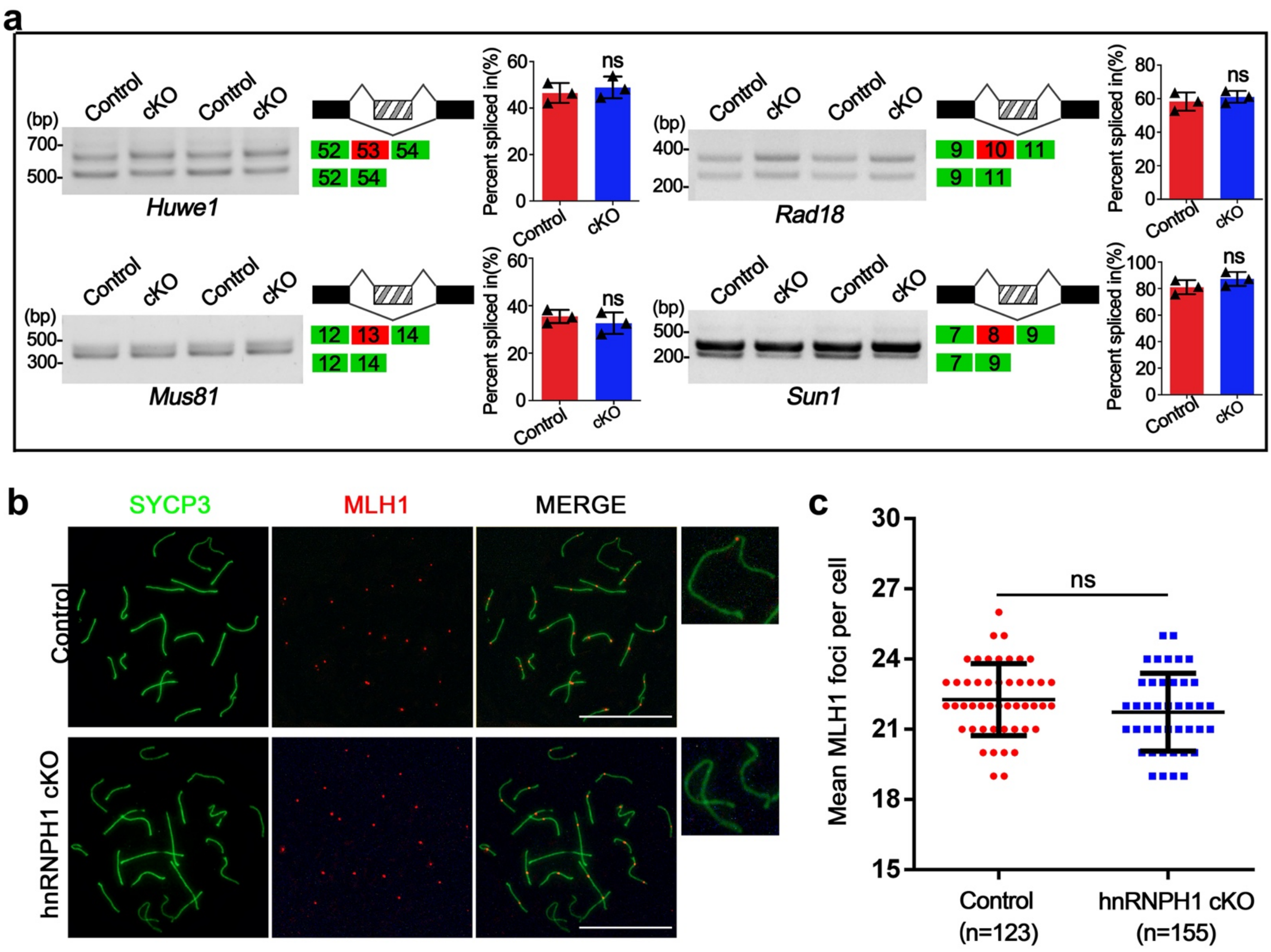

Supplementary Fig.9. Analyses of AS changes and MLH1 localization in hnRNPH1 cKO spermatocytes. a Representative examples of RT-PCR analyses for indicated AS events differentially regulated between control and hnRNPH1 cKO spermatocytes isolated from P25 mice are shown. Middle panels represent the schematic diagram of AS exons detected by RNA-Seq analysis. Right panels show the quantification of percent spliced in (PSI). Data are presented as mean ± SD, n = 3 (three biological replicates). A two-sided Student's *t*-test was performed. ns, not significant. Source data are provided as a source data file. **b** Co-immunofluorescence staining of SYCP3 with MLH1 in spermatocyte spreads at pachytene stage from control and hnRNPH1 cKO mice. Scale bar = 5 μm. **c** Mean MLH1 foci per cell in pachytene spermatocytes from the control and hnRNPH1 cKO mice. Data are presented as mean ± SD. A total of n = 675 control and n = 879 cKO spreads were counted from three biologically independent mice for each genotype. A two-tailed Mann–Whitney *U*-test was performed. ns, not significant. Source data are provided as a source data file.

Supplementary Figure 10

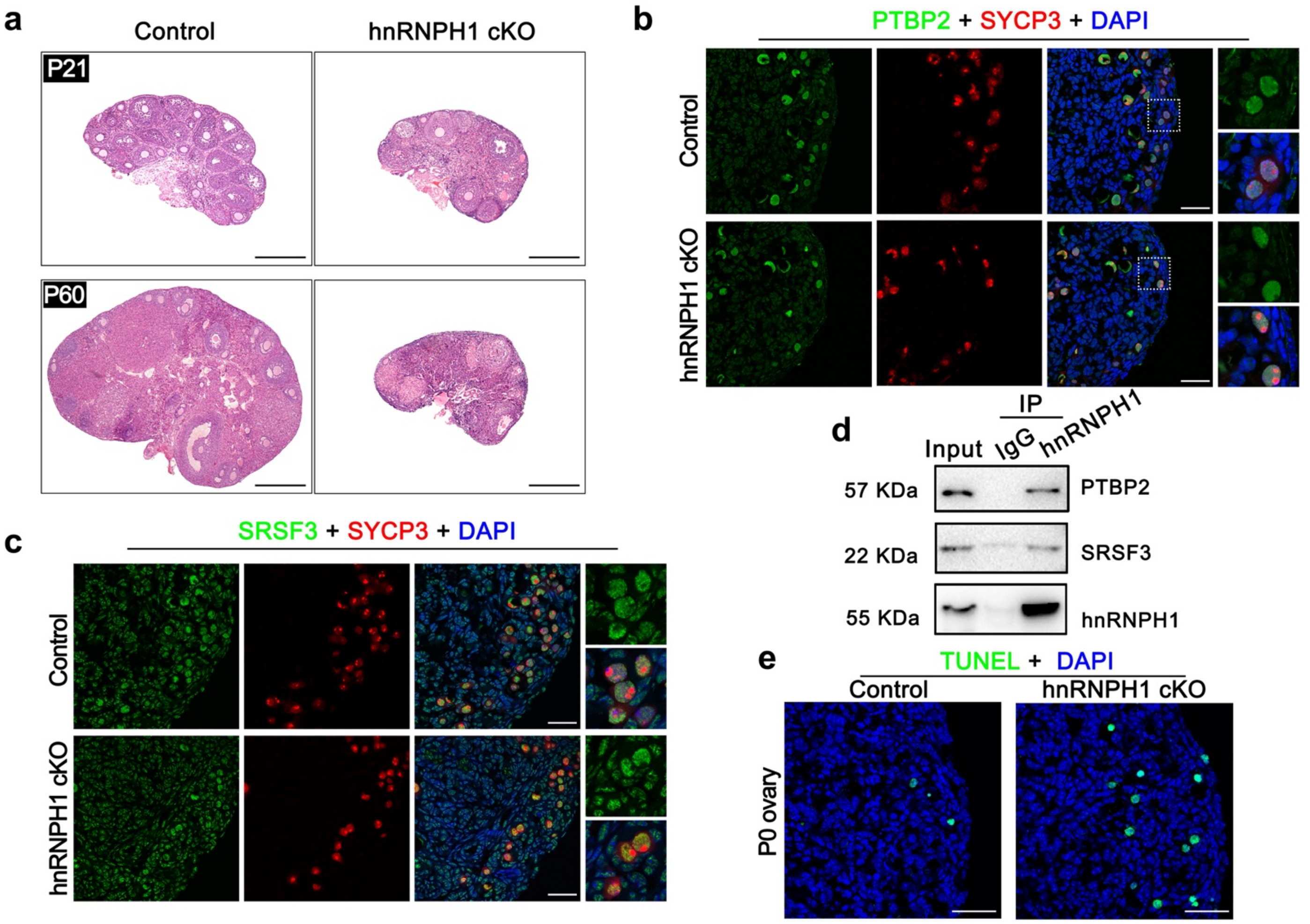

Supplementary Fig.10. hnRNPH1 is required for oogenesis and interacts with PTBP2 and SRSF3 in ovaries. a Representative micrographs of ovarian sections stained with hematoxylin and eosin (HE) from control and hnRNPH1 cKO mice at P21 and P60 are shown. Scale bars = 250μm. **b-c** Co-immunofluorescence staining of SYCP3 with PTBP2 (b) and SRSF3 (c) in ovaries from control and hnRNPH1 cKO mice at P0. The DNA was stained with DAPI. Scale bar = 50μm. **d** Validation of interactions between hnRNPH1 and two putative hnRNPH1-interacting proteins (PTBP2 and SRSF3) in mouse E17.5 ovaries by *in vivo* co-immunoprecipitation (Co-IP) assays are shown. IgG was used as a negative control. **e** Terminal deoxynucleotidyl transferase-mediated dUTP nick end labeling (TUNEL) staining of the ovaries derived from control and hnRNPH1 cKO mice at P0. The DNA was stained with DAPI. Scale bars = 50μm. Biologically independent mice (n = 3) for each genotype were examined (a-e). Source data are provided as a source data file.

Supplementary Figure 11

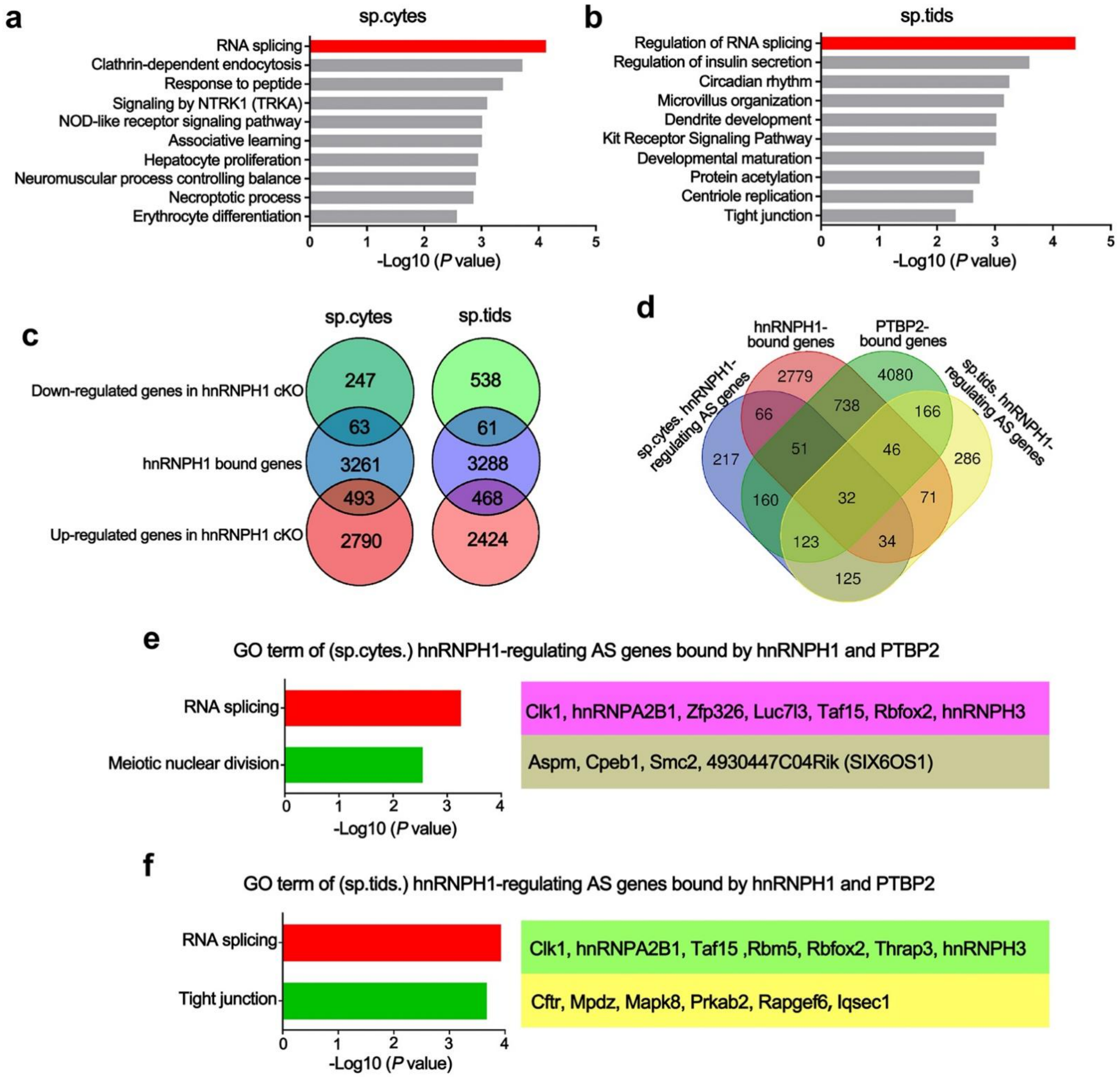

Supplementary Fig.11. GO term analyses of AS changed genes that bound by hnRNPH1 and comparison of hnRNPH1-regulated AS genes and PTBP2-bound genes. a-b GO term enrichment analyses of the overlapped genes between the hnRNPH1-bound and abnormal AS genes in hnRNPH1 cKO spermatocytes (a) or spermatids (b). **c** Venn diagrams showing overlap of hnRNPH1-bound genes, down-regulated genes, and up-regulated genes in hnRNPH1 cKO versus control spermatocytes (*left*) and spermatids (*right*). **d** Venn diagrams showing overlap of hnRNPH1-regulating AS genes in spermatocytes, hnRNPH1-regulating AS genes in spermatids, hnRNPH1-bound genes, and PTBP2 bound genes. **e-f** GO term enrichment analyses of hnRNPH1-regulating AS genes bound by both hnRNPH1 and PTBP2 in spermatocytes (e) and spermatids (f).

Supplementary Figure 12

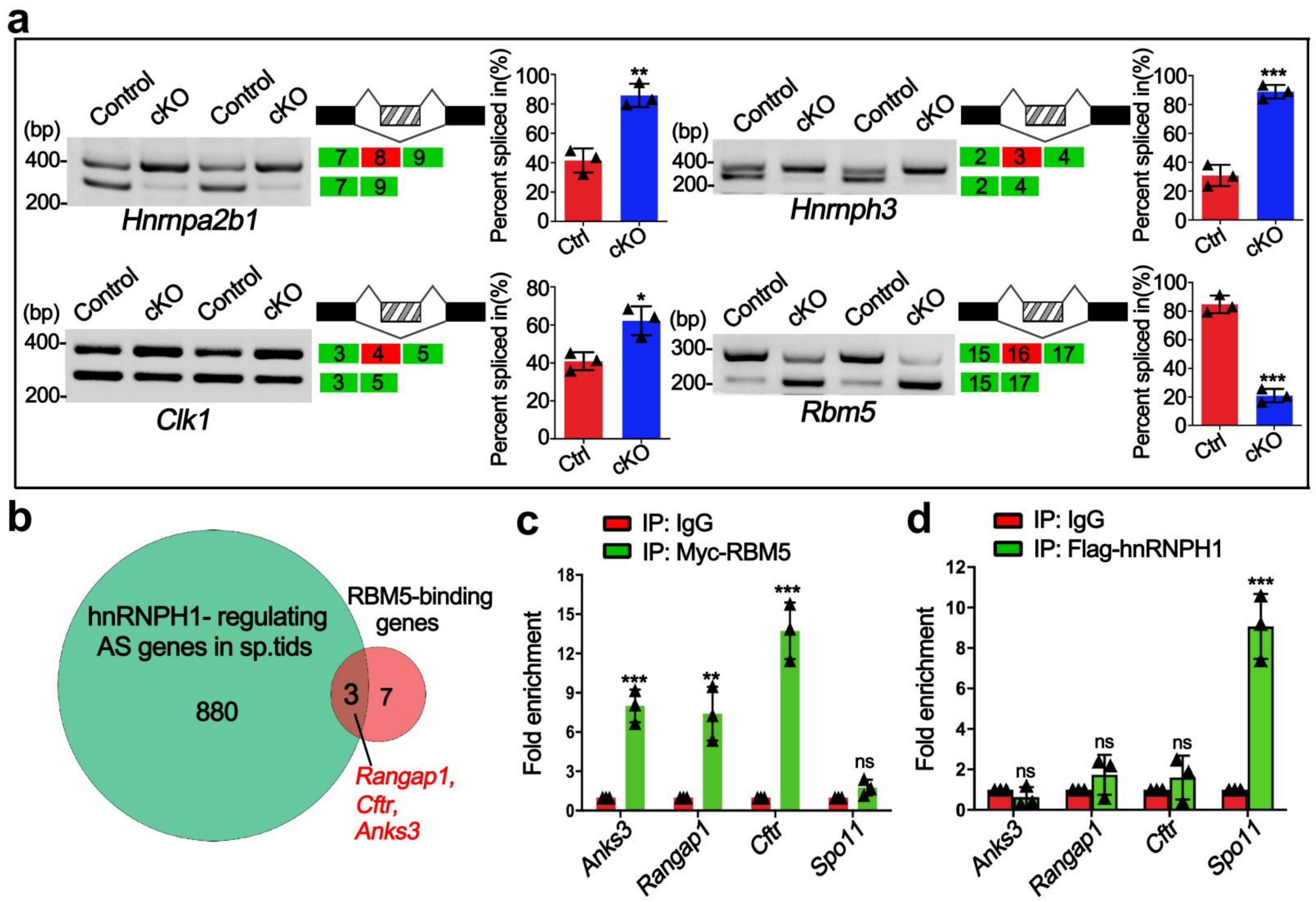

Supplementary Fig.12. RT-PCR analyses of AS changed genes regulated RNA splicing and RIP-qPCR assay of enrichment of *Rbm5* target genes to hnRNPH1. a Representative examples of RT-PCR analyses for indicated AS events differentially regulated between control and hnRNPH1 cKO spermatocytes. Middle panels represent the schematic diagram of alternatively spliced exons detected by RNA-Seq analysis. Right panels show the quantification of percent spliced in (PSI). Data are presented as mean ± SD, n = 3 (three biological replicates). A two-sided Student's *t*-test was performed, top left panel: ***p* =0.0026, top right panel: ****p* =0.0003, bottom left panel: **p* =0.0148, bottom right panel: **p* =0.0001. **b** Venn diagrams showing overlap of hnRNPH1-regulating AS genes in spermatids and RBM5-binding genes. **c-d** RIP-qPCR analyses for indicated mRNA of 4 genes co- precipitated by anti-Myc-RBM5 (c) and anti-Flag-hnRNPH1 (d) antibodies and control IgG in RIP experiments performed from purified germ cells isolated from WT mice at P25. Data are presented as mean ± SD, n = 3 (three biological replicates). A two-sided Student's *t*-test was performed, left to right: ****p* =0.0006, ***p* =0.0057, ****p* =0.0005, ****p* =0.0009. ns, not significant. Source data are provided as a source data file.

Supplementary Figure 13

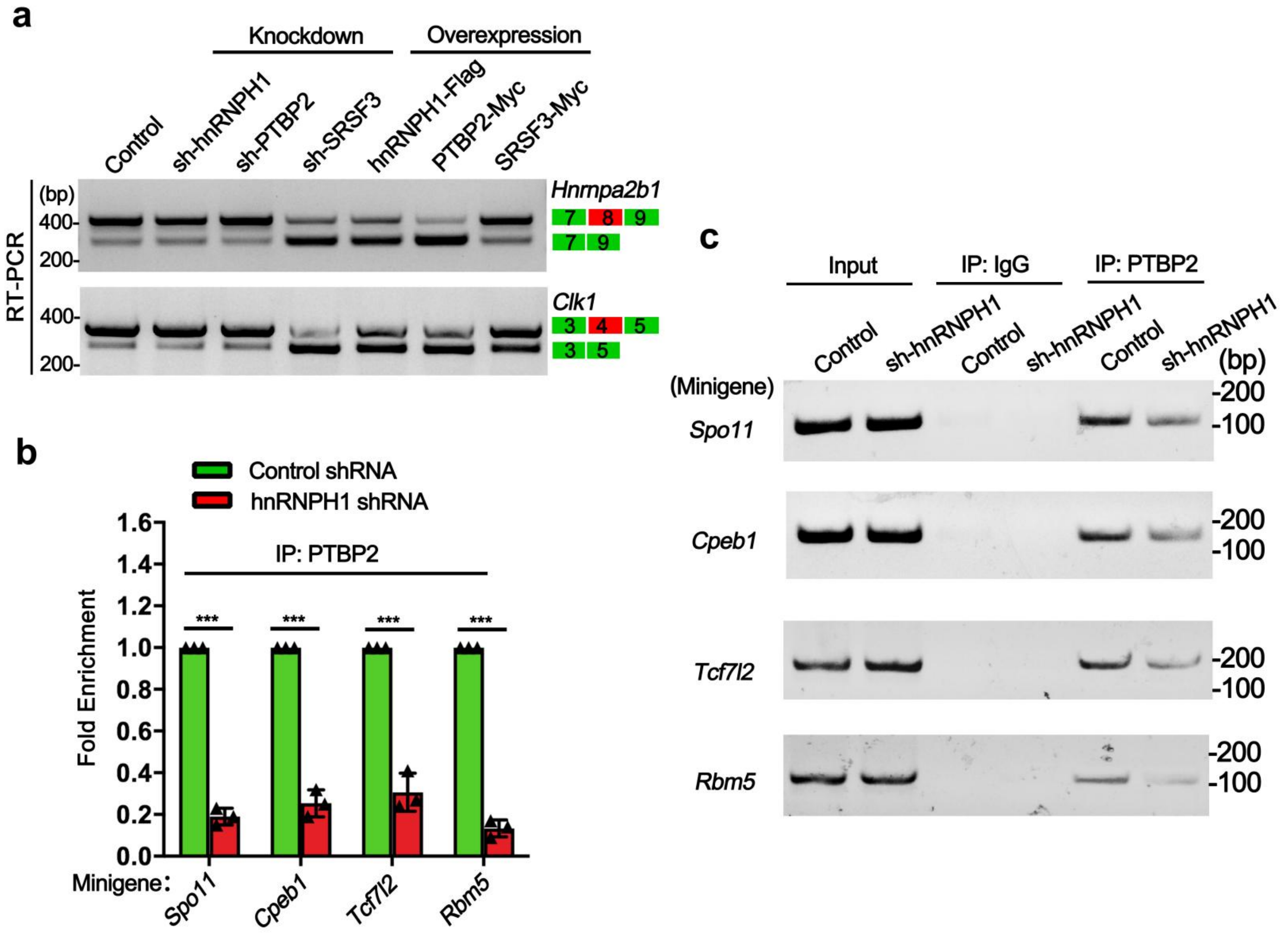

Supplementary Fig.13. RT-PCR analyses of AS upon overexpression and knockdown of indicated genes and the enrichment assay of 4 target minigenes to PTBP2. a RT-PCR analysis of splicing assay performed in HEK293T cells transfected with the indicated minigenes and knockdown/overexpression related vectors for hnRNPH1, PTBP2, and SRSF3. **b-c** RIP-qPCR(b) and RT-PCR (c) analyses for indicated mRNA of 4 genes co-precipitated by PTBP2-specific antibody in RIP experiments performed in HEK293T cells transfected with the indicated minigenes and knockdown related vectors for *Hnrnph1*. For (b), data are presented as mean ± SD, n = 3 (three biological replicates). A two-sided Student's *t*-test was performed, left to right: ****p* =4X10^{-6}, ****p* =3.8X10^{-5}, ****p* =0.0002, ****p* =3X10^{-6}. For (c), biologically independent experiment (n = 3) was examined. Source data are provided as a source data file.

Supplementary Figure 14

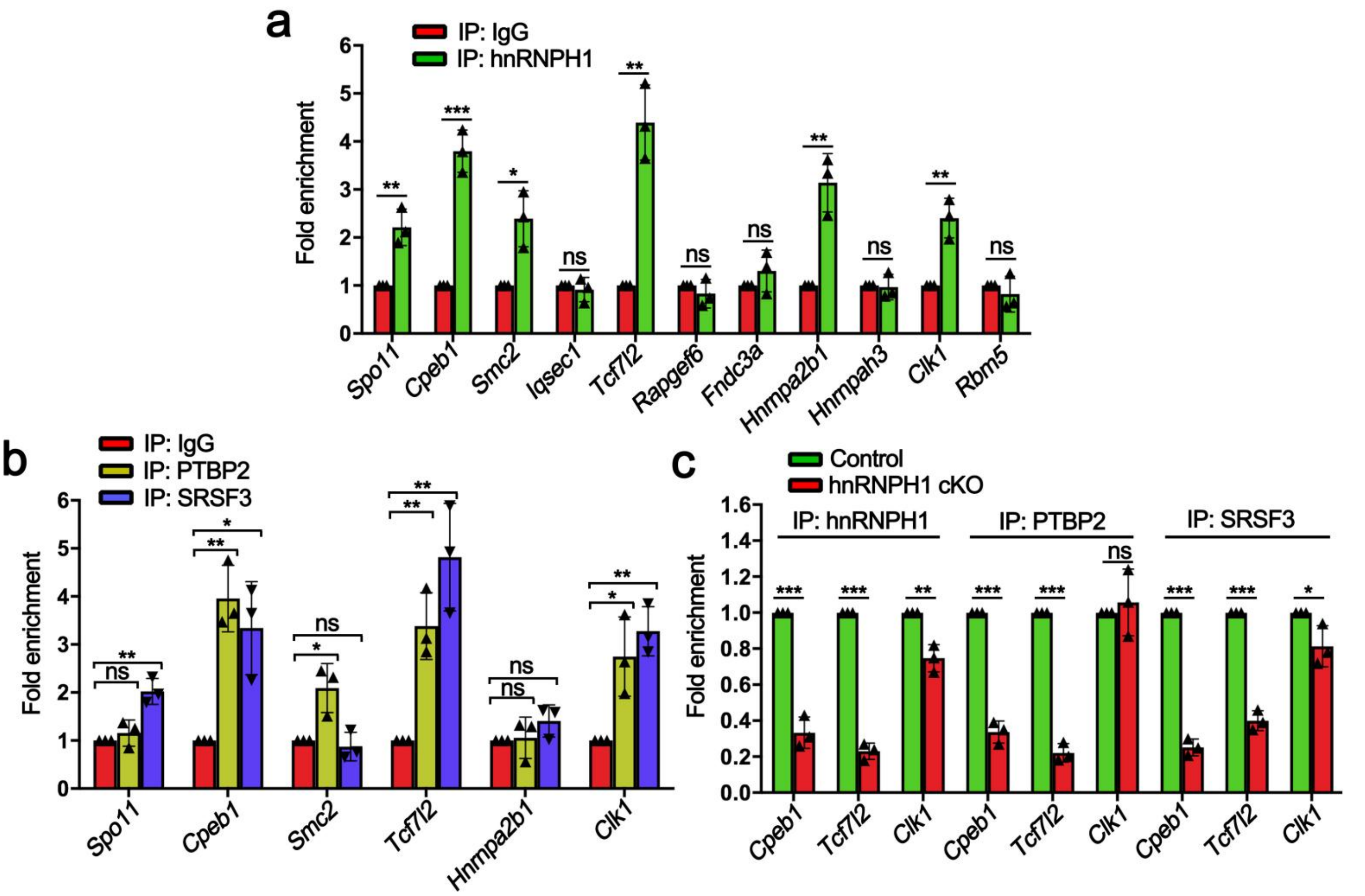

Supplementary Fig.14. hnRNPH1 cooperates with PTBP2 and SRSF3 to regulate mRNA splicing in ovaries. a Histograms show RIP-qPCR analyses of selected 11 gene mRNAs precipitated by anti-hnRNPH1 antibody and control IgG in P0 ovaries. Data are presented as mean ± SD, n = 3 (three biological replicates). A two-sided Student's *t*-test was performed, left to right: **p =0.0052, ***p =0.0004, *p =0.0143, **p =0.0017, **p =0.0036, **p =0.0041, ns, not significant. **b** Histograms show RIP-qPCR analyses of selected 6 gene mRNAs precipitated by anti-PTBP2, anti-SRSF3 antibodies and control IgG in P0 ovaries. Data are presented as mean ± SD, n = 3 (three biological replicates). A two-sided Student's *t*-test was performed, left to right: **p =0.0028, **p =0.0018, *p =0.0137, *p =0.0205, **p =0.0040, **p =0.0041, *p =0.0215, **p =0.0015, ns, not significant. **c** RIP-qPCR analyses of the association of the selected 3 gene mRNAs with PTBP2 and SRSF3 in control and hnRNPH1 cKO ovaries are shown. Data are presented as mean ± SD, n = 3 (three biological replicates). A two-sided Student's *t*-test was performed, left to right: ***p =0.0002, ***p =8X10^{-6}, **p =0.0043, ***p =4.7X10^{-5}, ***p =1.2X10^{-5}, ***p =1X10^{-5}, ***p =4.9X10^{-5}, *p =0.0467, ns, not significant. Source data are provided as a source data file.
