# Supplementary material for: hnRNPH1 recruits PTBP2 and SRSF3 to modulate alternative splicing in germ cells
Source: Nat Commun. 2022 Jun 23;13:3588. doi: 10.1038/s41467-022-31364-7 (PMC9226075; doi:10.1038/s41467-022-31364-7)
Supplement: Supplementary file 3 — Description of Additional Supplementary Files [file 41467_2022_31364_MOESM3_ESM.pdf]

Description of Additional Supplementary Files

File name: Supplementary Data 1
Description: Identification of hnRNPH1 interactors by mass spectrometry.

File name: Supplementary Data 2
Description: Total identified abnormal alternative splicing genes in *hnRNPH1* cKO pachytene spermatocytes by RNA-Seq. To detect valid alternative splicing events, those with false discovery rate (FDR) < 0.05 and |△PSI|> 10% were categorized as differential alternative splicing events. A two-sided likelihood-ratio test was performed and adjustments were made for multiple comparisons.

File name: Supplementary Data 3
Description: Total identified abnormal alternative splicing genes in *hnRNPH1* cKO round spermatids by RNA-Seq. Total identified abnormal alternative splicing genes in *hnRNPH1* cKO pachytene spermatocytes by RNA-Seq. To detect valid alternative splicing events, those with false discovery rate (FDR) < 0.05 and |△PSI|> 10% were categorized as differential alternative splicing events. A two-sided likelihood-ratio test was performed and adjustments were made for multiple comparisons.

File name: Supplementary Data 4
Description: Total identified differentially expressed genes between *hnRNPH1* cKO and control pachytene spermatocytes by RNA-Seq.

File name: Supplementary Data 5
Description: Total identified differentially expressed genes between hnRNPH1 cKO and control round spermatids by RNA-Seq.

File name: Supplementary Data 6
Description: Total identified abnormal alternative splicing genes in *Ptbp2* cKO testes by analysis of published RNA-Seq data. Total identified abnormal alternative splicing genes in *hnRNPH1* cKO pachytene spermatocytes by RNA-Seq. To detect valid alternative splicing events, those with false discovery rate (FDR) < 0.05 and |△PSI|> 10% were categorized as differential alternative splicing events. A two-sided likelihood-ratio test was performed and adjustments were made for multiple comparisons.

File name: Supplementary Data 7
Description: Total identified hnRNPH1-binding target genes by analysis of published ChIP-seq data. A two-sided likelihood-ratio test was performed and adjustments were made for multiple comparisons.

File name: Supplementary Data 8

Description: Total identified hnRNPH1-binding target genes by RIP-seq using isolated germ cells of P28 testes.

File name: Supplementary Data 9

Description: Primer sequences are used in this study.

File name: Supplementary Data 10

Description: Antibodies used in this study.
