# Supplementary material for: hnRNPH1 recruits PTBP2 and SRSF3 to modulate alternative splicing in germ cells
Source: Nat Commun. 2022 Jun 23;13:3588. doi: 10.1038/s41467-022-31364-7 (PMC9226075; doi:10.1038/s41467-022-31364-7)
Supplement: Supplementary file 14 — Reporting Summary [file 41467_2022_31364_MOESM14_ESM.pdf]

nature portfolio

Corresponding author(s): Shuiqiao Yuan

Last updated by author(s): May 30, 2022

Reporting Summary

Nature Portfolio wishes to improve the reproducibility of the work that we publish. This form provides structure for consistency and transparency in reporting. For further information on Nature Portfolio policies, see our Editorial Policies and the Editorial Policy Checklist.

Statistics

For all statistical analyses, confirm that the following items are present in the figure legend, table legend, main text, or Methods section.

n/a	Confirmed	
☐	☒	The exact sample size (*n*) for each experimental group/condition, given as a discrete number and unit of measurement
☐	☒	A statement on whether measurements were taken from distinct samples or whether the same sample was measured repeatedly
☐	☒	The statistical test(s) used AND whether they are one- or two-sided<br>*Only common tests should be described solely by name; describe more complex techniques in the Methods section.*
☐	☒	A description of all covariates tested
☐	☒	A description of any assumptions or corrections, such as tests of normality and adjustment for multiple comparisons
☐	☒	A full description of the statistical parameters including central tendency (e.g. means) or other basic estimates (e.g. regression coefficient) AND variation (e.g. standard deviation) or associated estimates of uncertainty (e.g. confidence intervals)
☐	☒	For null hypothesis testing, the test statistic (e.g. *F*, *t*, *r*) with confidence intervals, effect sizes, degrees of freedom and *P* value noted<br>*Give P values as exact values whenever suitable.*
☒	☐	For Bayesian analysis, information on the choice of priors and Markov chain Monte Carlo settings
☒	☐	For hierarchical and complex designs, identification of the appropriate level for tests and full reporting of outcomes
☒	☐	Estimates of effect sizes (e.g. Cohen's *d*, Pearson's *r*), indicating how they were calculated

Our web collection on statistics for biologists contains articles on many of the points above.

Software and code

Policy information about availability of computer code

Data collection	Confocal data was collected using Leica Application suiteX LAS X software. RNA-seq and ChIP libraries were subjected to pair-end sequencing on Illumina HiSeq 2500. and Illumina Hi-Seq 2000, respectively.
Data analysis	GraphPad Prism 8.0, Integrative Genomics Viewer (IGV), Image 1.52, UCSC mm10, Trimmomatic (0.39) were used for data analysis.

For manuscripts utilizing custom algorithms or software that are central to the research but not yet described in published literature, software must be made available to editors and reviewers. We strongly encourage code deposition in a community repository (e.g. GitHub). See the Nature Portfolio guidelines for submitting code & software for further information.

Data

Policy information about availability of data

All manuscripts must include a data availability statement. This statement should provide the following information, where applicable:

- Accession codes, unique identifiers, or web links for publicly available datasets
- A description of any restrictions on data availability
- For clinical datasets or third party data, please ensure that the statement adheres to our policy

All data needed to evaluate the conclusions in the paper are present in the article and/or the Supplementary Materials. All RNA-seq and RIP-seq data are deposited in the NCBI SRA (Sequence Read Achieve) database with the accession number of PRJNA771927. Public ChIP-seq data sets used in this study are also accessible through GEO Series accession number of PRJNA491668. The String database (https://cn.string-db.org/) was used for analysis of protein-protein interaction network. The source data are provided in Supplementary Figs 15-18 and as a source data file. The authors declare that all data supporting the findings of this study are available within the article and its supplementary information files or from the corresponding author upon reasonable request. Source data are provided with this paper.

Field-specific reporting

Please select the one below that is the best fit for your research. If you are not sure, read the appropriate sections before making your selection.

☒ Life sciences ☐ Behavioural & social sciences ☐ Ecological, evolutionary & environmental sciences

For a reference copy of the document with all sections, see nature.com/documents/nr-reporting-summary-flat.pdf

Life sciences study design

All studies must disclose on these points even when the disclosure is negative.

Sample size	No statistical method was used to predetermine sample size. We followed the conventional way of quantification accepted in our published paper (PMID: 31624244, 33674260, and 34815802) to determine the sample size.
Data exclusions	No data were excluded from the analysis.
Replication	Each conclusion in the manuscript was based on results that were reproduced in at least three independent experiments and in at least three independent mice of each genotype. The replicate data were reliable.
Randomization	Mice were categorized based on their genotypes. The genotypes were determined by PCR. For experiments other than those involving mice, samples were non-randomly chosen according to the genotype.
Blinding	The investigators were not blinded to allocation during the experiments or to outcome assessment.<br>This is because the phenotypes were quite obvious that observer can be sure without blind test.<br>Further, the observer unbiasedly and carefully performed the quantification with enough sample number to make sure the conclusion.

Reporting for specific materials, systems and methods

We require information from authors about some types of materials, experimental systems and methods used in many studies. Here, indicate whether each material, system or method listed is relevant to your study. If you are not sure if a list item applies to your research, read the appropriate section before selecting a response.

Materials & experimental systems

n/a	Involved in the study	
☐	☒	Antibodies
☐	☒	Eukaryotic cell lines
☒	☐	Palaeontology and archaeology
☐	☒	Animals and other organisms
☒	☐	Human research participants
☒	☐	Clinical data
☒	☐	Dual use research of concern

Methods

n/a	Involved in the study	
☐	☒	ChIP-seq
☒	☐	Flow cytometry
☒	☐	MRI-based neuroimaging

Antibodies

Antibodies used	Supplementary Data 10 provided with manuscript contains information on all antibodies used in the study.
Validation	All antibodies were validated by the manufacturer and the detailed dilution ratio was provided in the supplementary Data 10.<br>Antibody specificity was evaluated using the proper negative controls.

Eukaryotic cell lines

Policy information about cell lines

Cell line source(s)	HEK293T cells (Fig.2f-i, Fig.8e and Supplementary Fig.12c-d, and Fig.13) used in this study were obtained from Stem Cell Bank at Chinese Academy of Sciences. More information in Methods section.
Authentication	For all cell lines used in the manuscript, cell line identity was authenticated by supplier with short tandem repeat profiling .
Mycoplasma contamination	We confirm that our cell line has no mycoplasma contamination.
Commonly misidentified lines<br>(See ICLAC register)	No cell lines used are listed in the database of commonly misidentified cell lines.

Animals and other organisms

Policy information about studies involving animals; ARRIVE guidelines recommended for reporting animal research

Laboratory animals	The Hnrnph1+/flox mice were obtained by chimera formation and germline transmission. 6 week-old mMice were then crossed with FLP transgenic mice to remove the neomycin cassette and maintained on a C57BL/6J background. Stra8-GFPCre mouse line in the C57BL/6J background was obtained from Dr. Minghan Tong's Laboratory at Center for Excellence in Molecular Cell Science, Chinese Academy of Sciences. 6 week-old Stra8-GFPCre males were first crossed with 6 week-old Hnrnph1flox/flox females to generate the Stra8-GFPCre; Hnrnph1+/flox males, then the 8 week-oldadult Stra8-GFPCre; Hnrnph1+/flox male mice were bred with Hnrnph1flox/flox female mice to obtain the Stra8-GFPCre; Hnrnph1flox/△ (designated as hnRNPH1 cKO) male and female mice. For the global Hnrnph1 knockout mouse generation, Hnrnph1flox/△ male mice were bred with Hnrnph1flox/△ female mice to generate Hnrnph1△/△ mice (designated as hnRNPH1 gKO). All mice were maintained in ambient room temperature (22°C°C) with humidity of 40–70% and light/dark cycle of 12h/12h.
Wild animals	This study did not involve wild animals.
Field-collected samples	This study did not involve samples collected from the field.
Ethics oversight	All the experimental animal procedures were approved by the Institutional Animal Care and Use Committee of Tongji Medical College, Huazhong University of Science and Technology, and the mice were fed in specific pathogen-free facilities of Huazhong University of Science and Technology.

Note that full information on the approval of the study protocol must also be provided in the manuscript.

ChIP-seq

Data deposition

☒ Confirm that both raw and final processed data have been deposited in a public database such as GEO.

☒ Confirm that you have deposited or provided access to graph files (e.g. BED files) for the called peaks.

Data access links<br>*May remain private before publication.*	https://www.ncbi.nlm.nih.gov/sra?term=SRP162019
Files in database submission	Supplementary Fig 8 and Data 7
Genome browser session<br>(e.g. UCSC)	UCSC mm10

Methodology

Replicates	Two replicates for IP and input samples were used.
Sequencing depth	Sequencing depth for each experiment is 3G, the total number of clean reads is 24658216~30843852, uniquely mapped reads is about 20%, length of reads is 150bp and they were paired end.
Antibodies	Anti-hnRNPH1 antibody form Aviva, catalog number: ARP58479
Peak calling parameters	Peak calling was performed by MACS2 (http://github. com/taoliu/MACS), with input used as the control. For, MACS2 default parameters with broad peak option and a broad -cutoff of 0.05 (P value) were used.
Data quality	FastQC was used detect the data quality for the raw data and clean data, including quality distribution, balancing analysis of base.
Software	The software of FastQC (version v0.11.5), Trimmomatic (version 0,36), STAR (version v2.5.3a), RSeQC (version 2.6), MACS2 (version 2.1.1), Homer (version v4.10), deepTools (version 2.4.1) and ChIPseeker (version1.5.1) were used to analyze the ChIP-Seq data in this study.
