# Supplementary material for: hnRNPH1 recruits PTBP2 and SRSF3 to modulate alternative splicing in germ cells
Source: Nat Commun. 2022 Jun 23;13:3588. doi: 10.1038/s41467-022-31364-7 (PMC9226075; doi:10.1038/s41467-022-31364-7)

**Original un-cropped, raw scans of Western blot membranes correspond to Figure 3c, Figure 5c, Figure 6b, and Figure 8e**


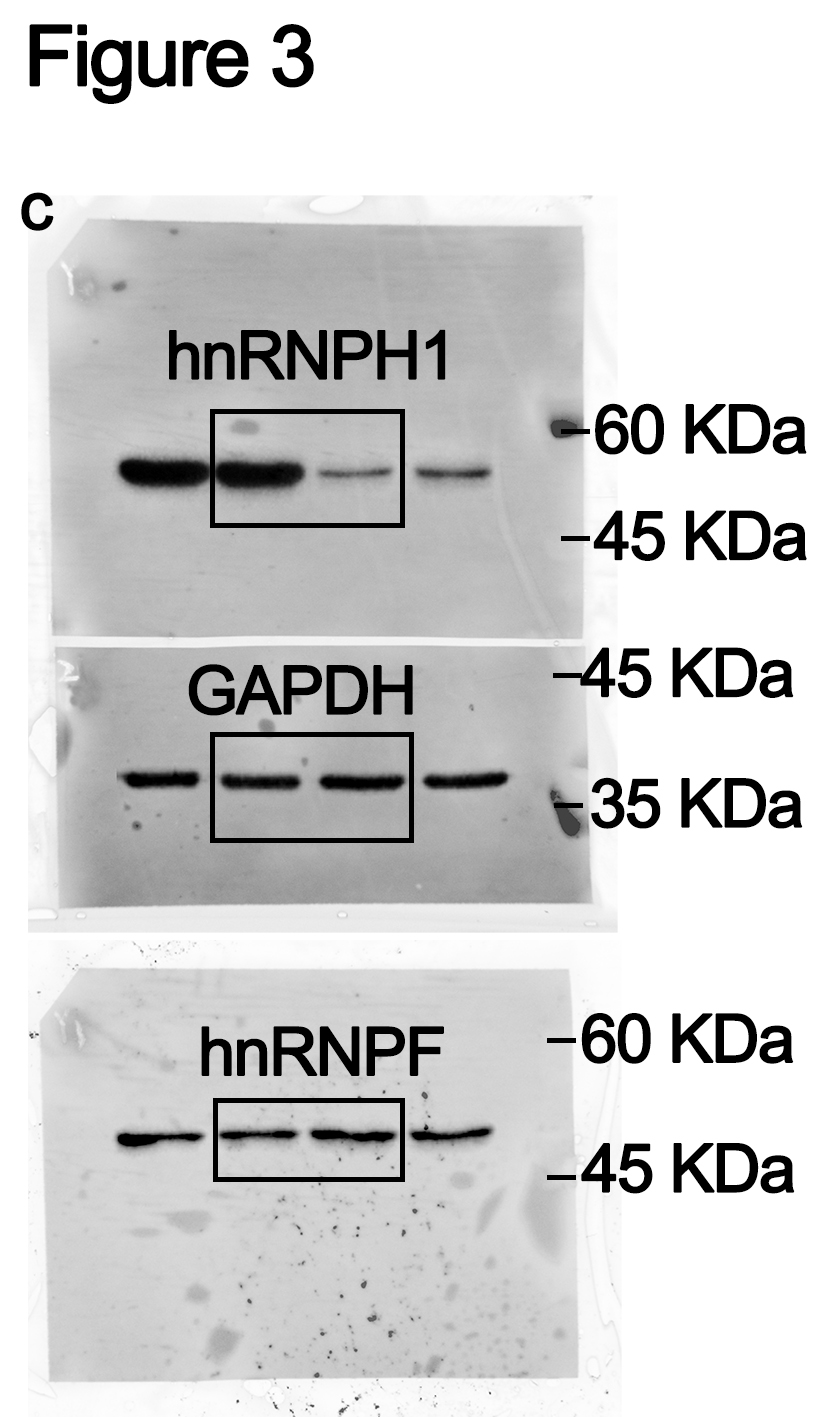


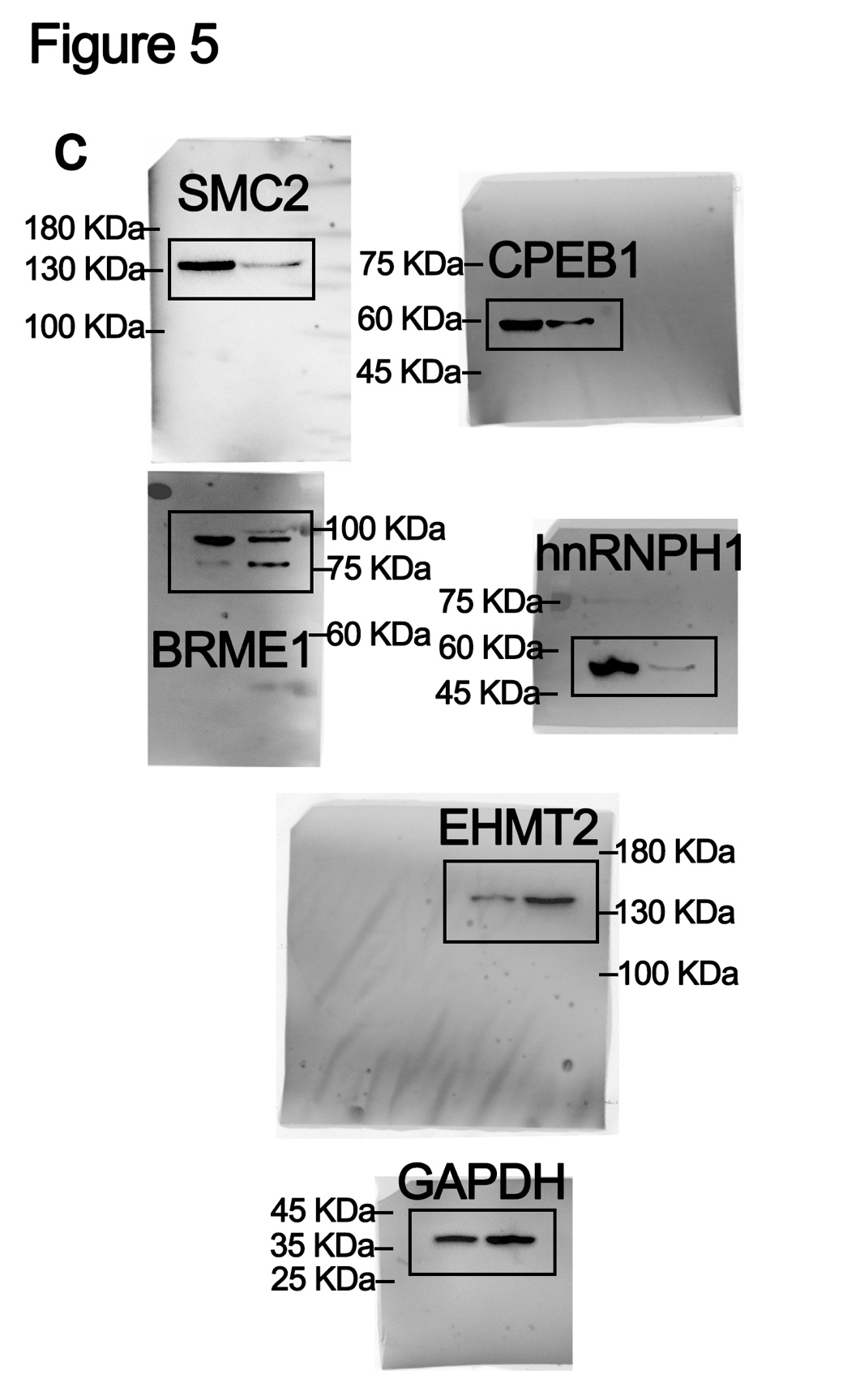


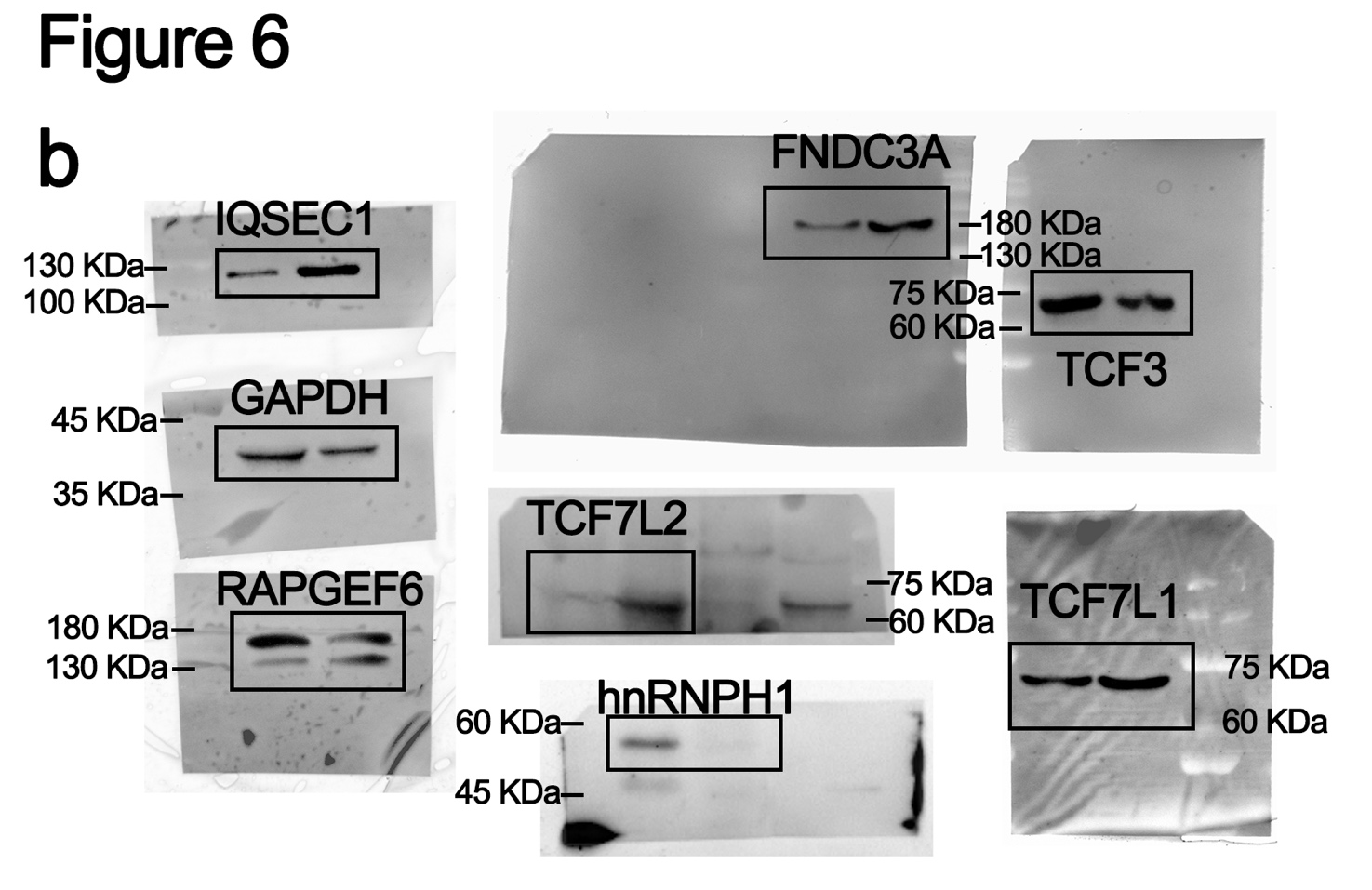


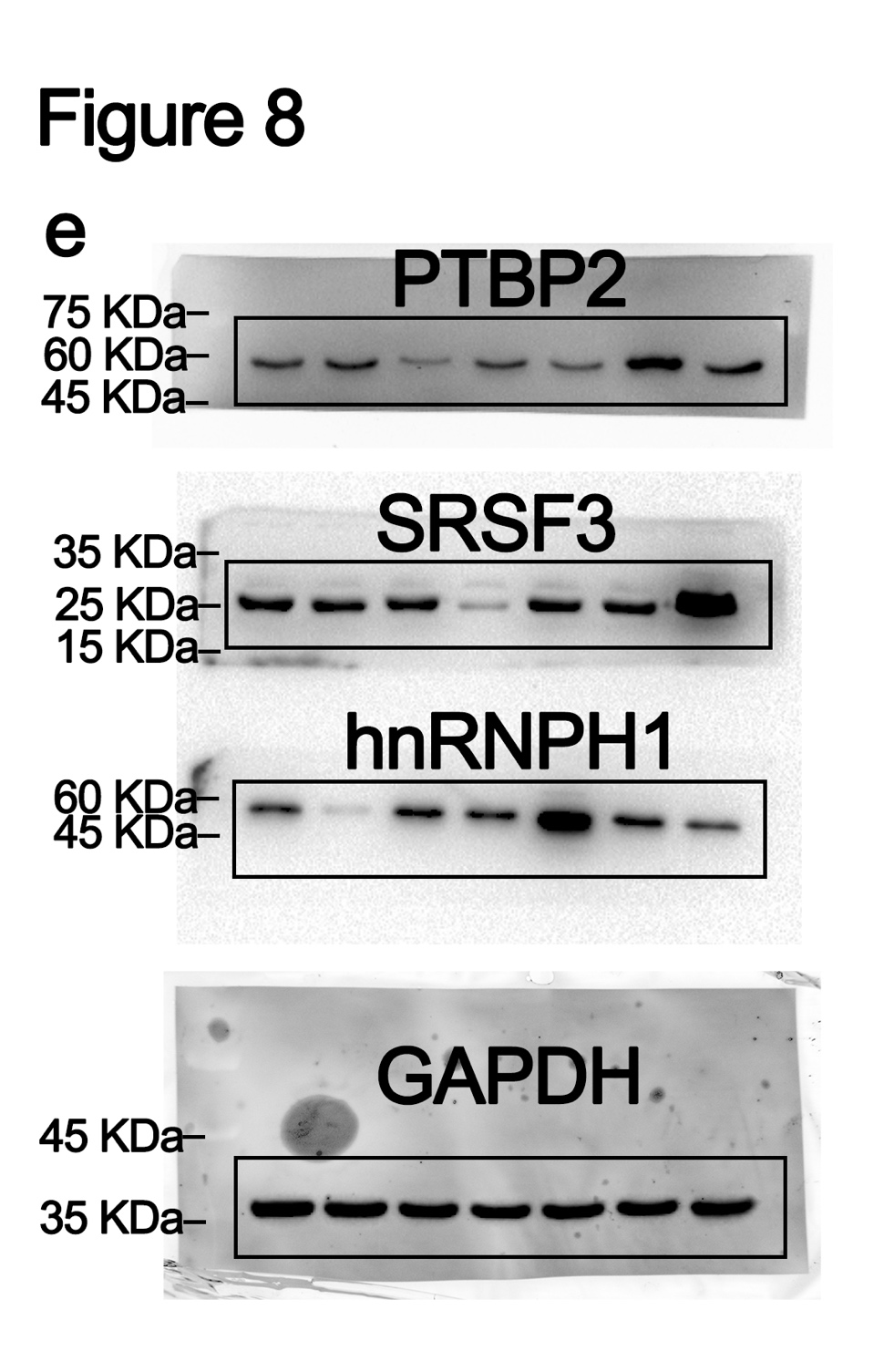

Supplement: Supplementary file 15 — Source Data [file 41467_2022_31364_MOESM15_ESM.zip › Source data/Uncropped WB-Fig.3c,5c,6b,8e.docx]
