# Supplementary figures and images for: hnRNPH1 recruits PTBP2 and SRSF3 to modulate alternative splicing in germ cells
Source: Nat Commun. 2022 Jun 23;13:3588. doi: 10.1038/s41467-022-31364-7 (PMC9226075; doi:10.1038/s41467-022-31364-7)

**Original un-cropped, raw scans of Western blot membranes correspond to Figure 2d, f-i.**


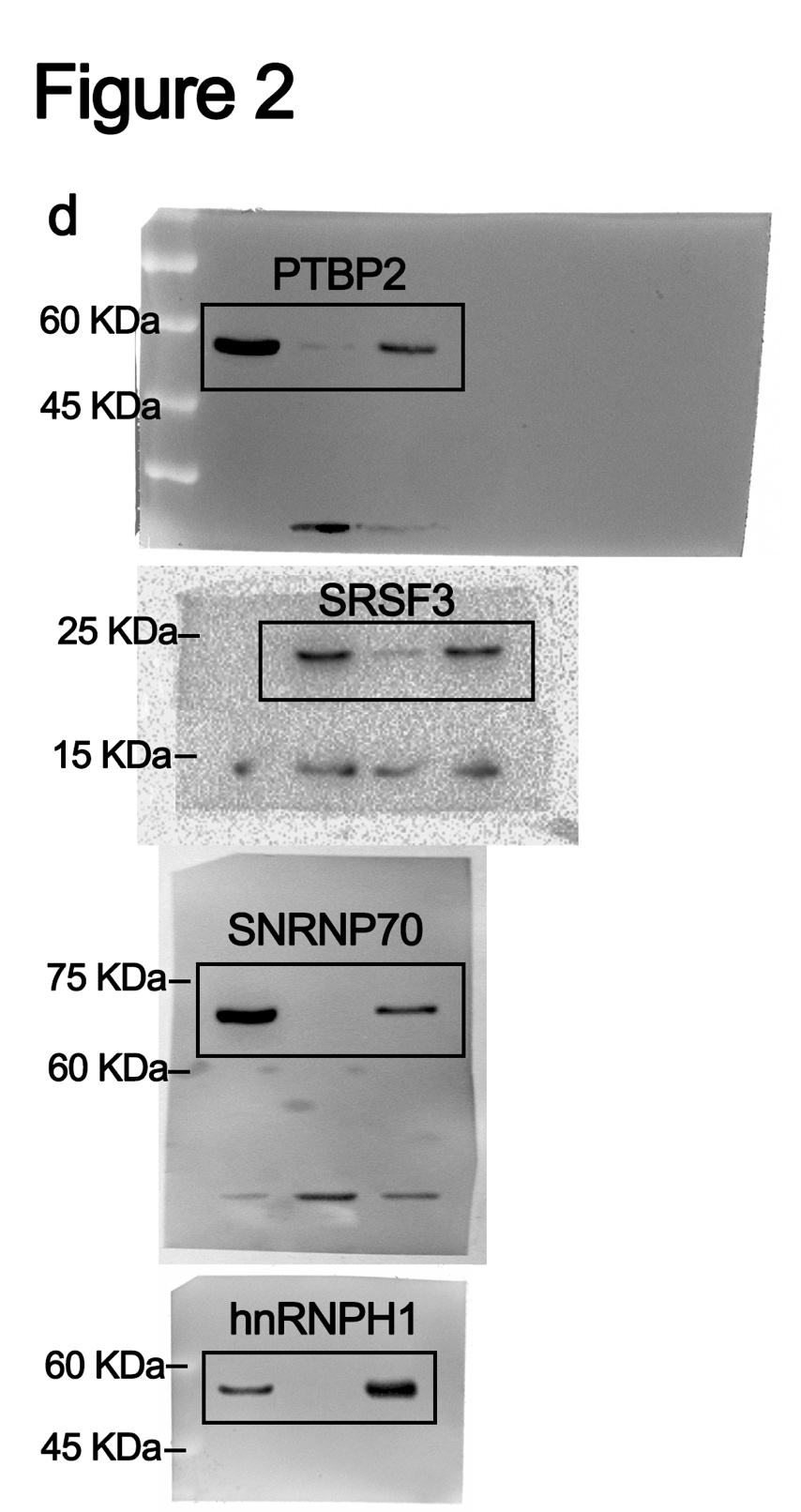


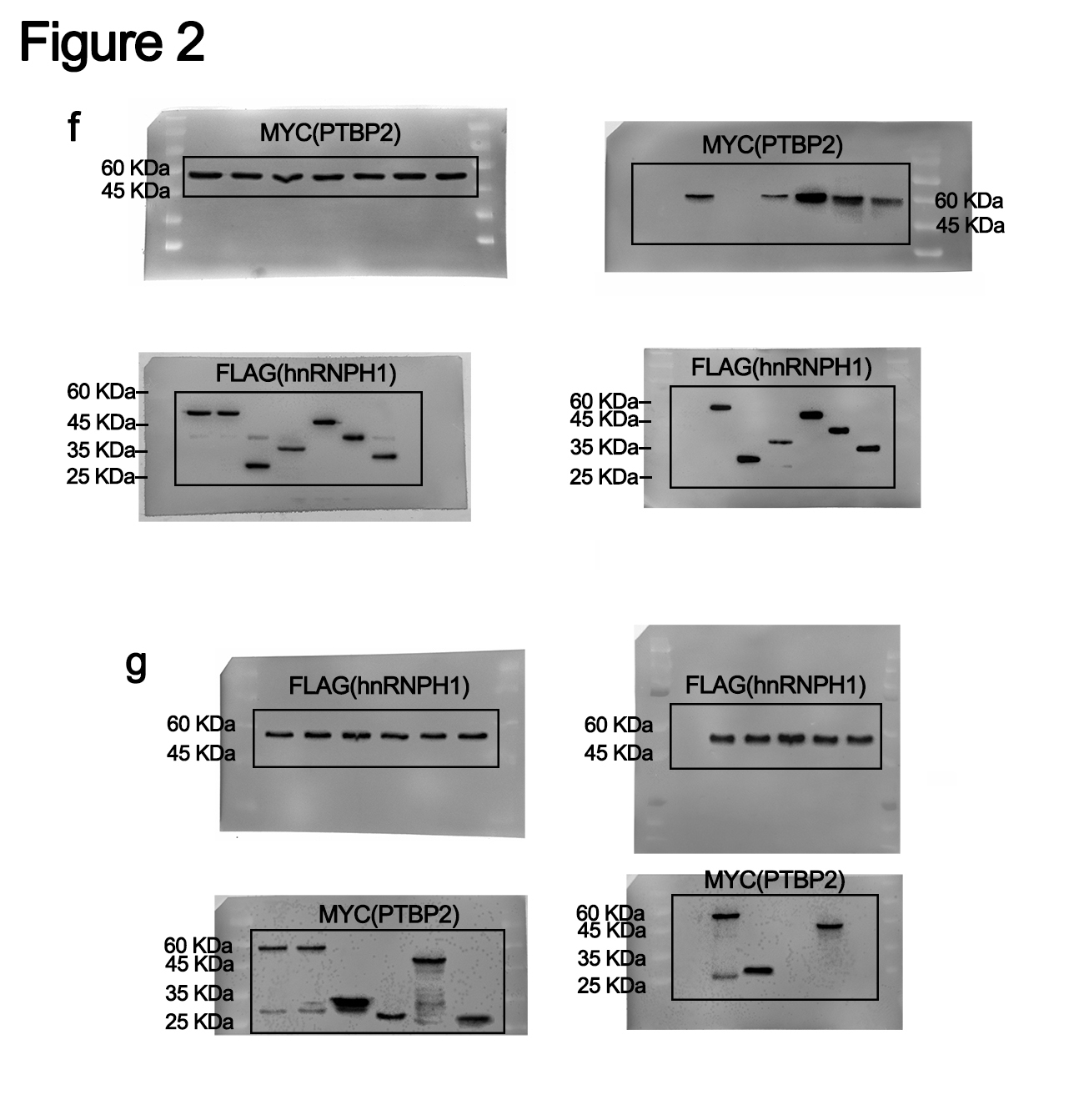


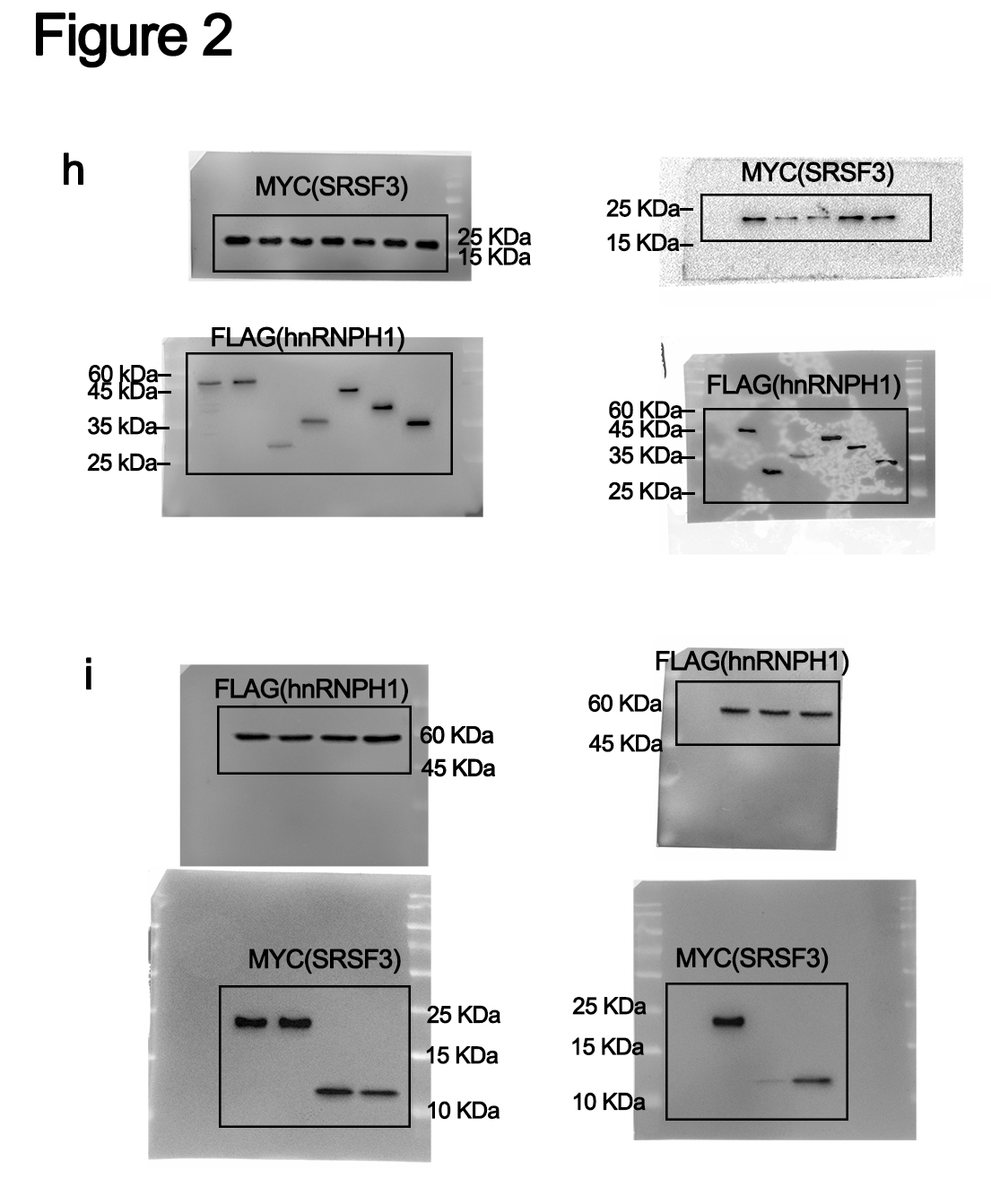

Supplement: Supplementary file 15 — Source Data [file 41467_2022_31364_MOESM15_ESM.zip › Source data/Uncropped WB-Fig.2d, f-i.docx]

**Original un-cropped, raw scans of RT-PCR correspond to Figure 9b and Supplementary Figure 13c.**


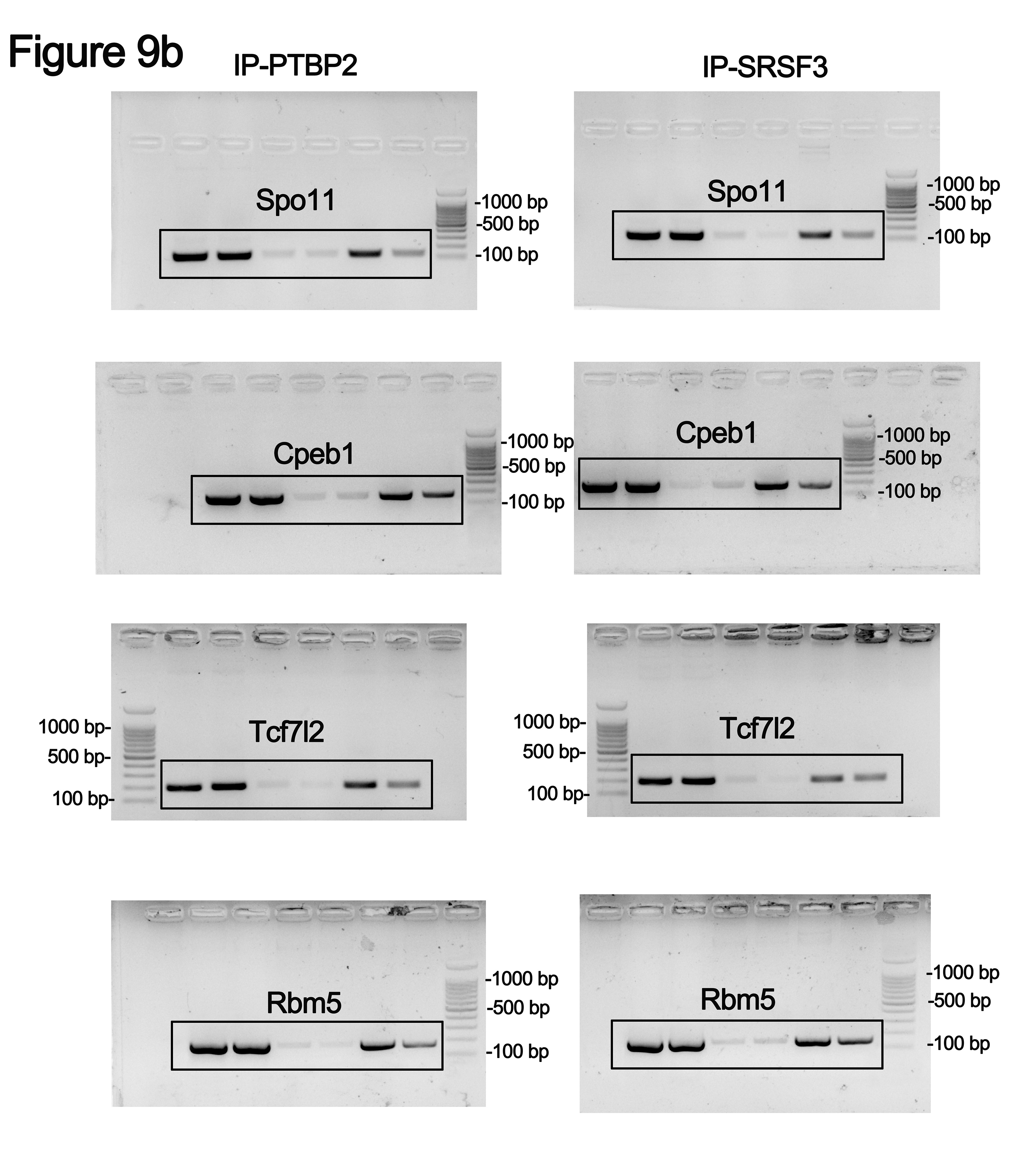


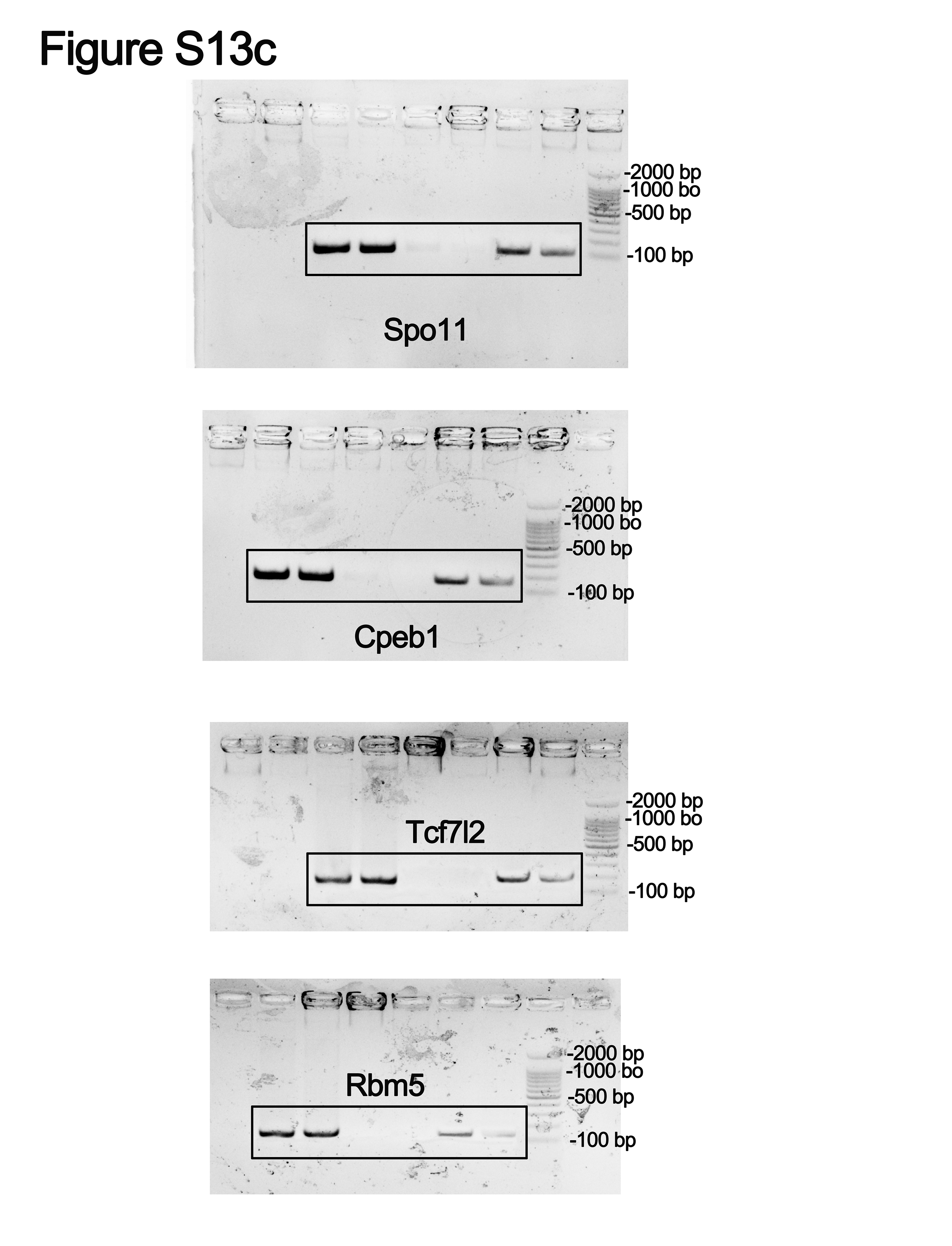

Supplement: Supplementary file 15 — Source Data [file 41467_2022_31364_MOESM15_ESM.zip › Source data/un-cropped RT-PCR gel.docx]
